# The NO/ONOO-Cycle as the Central Cause of Heart Failure

**DOI:** 10.3390/ijms141122274

**Published:** 2013-11-13

**Authors:** Martin L. Pall

**Affiliations:** Biochemistry and Basic Medical Sciences, Washington State University, 638 NE 41st Ave., Portland, OR 97232-3312, USA; E-Mail: martin_pall@wsu.edu; Tel.: +1-503-232-3883

**Keywords:** nitrosative stress, reactive oxygen/nitrogen species, free radicals, inflammatory biochemistry, mitochondrial dysfunction, Ca^2+^ receptors, peroxynitrite, tetrahydrobiopterin oxidation

## Abstract

The NO/ONOO-cycle is a primarily local, biochemical vicious cycle mechanism, centered on elevated peroxynitrite and oxidative stress, but also involving 10 additional elements: NF-κB, inflammatory cytokines, iNOS, nitric oxide (NO), superoxide, mitochondrial dysfunction (lowered energy charge, ATP), NMDA activity, intracellular Ca^2+^, TRP receptors and tetrahydrobiopterin depletion. All 12 of these elements have causal roles in heart failure (HF) and each is linked through a total of 87 studies to specific correlates of HF. Two apparent causal factors of HF, RhoA and endothelin-1, each act as tissue-limited cycle elements. Nineteen stressors that initiate cases of HF, each act to raise multiple cycle elements, potentially initiating the cycle in this way. Different types of HF, left *vs*. right ventricular HF, with or without arrhythmia, *etc*., may differ from one another in the regions of the myocardium most impacted by the cycle. None of the elements of the cycle or the mechanisms linking them are original, but they collectively produce the robust nature of the NO/ONOO-cycle which creates a major challenge for treatment of HF or other proposed NO/ONOO-cycle diseases. Elevated peroxynitrite/NO ratio and consequent oxidative stress are essential to both HF and the NO/ONOO-cycle.

## 1. Introduction

The NO/ONOO-cycle is a primarily local, biochemical vicious cycle, which depending on where it is located in the body, may be the cause of various chronic inflammatory diseases [1–8]. Various diseases have been argued to be possible NO/ONOO-cycle diseases, and, for many of them, the cases made have been relatively superficial. Most of these possible NO/ONOO-cycle diseases are probably located primarily in regions of the central nervous system [2–8]. However, a recent, very detailed case, argues that pulmonary arterial hypertension is a probable NO/ONOO-cycle disease [1], suggesting that other types of cardiovascular diseases may also be candidates for being caused by this mechanism.

The current paper explores the possibility that heart failure (HF) may be a NO/ONOO-cycle disease. It is based on findings that each of the 12 elements of the cycle is elevated in HF and that each of them has causal roles in HF. The cycle is centered on the elevation of peroxynitrite (ONOO-), a potent oxidant, and on consequent oxidative stress, making the possible cycle mechanism of HF relevant to this special issue of the journal. The possible causation of HF by the cycle is also supported by findings that various stressors reported to initiate cases of HF act to raise cycle elements and therefore may be able to initiate the cycle by raising such elements. However, in order to assess the strength of these and other arguments regarding the possible etiology of HF, it is necessary to look at the mechanisms of the NO/ONOO-cycle and the consequent predicted properties of cycle-caused diseases.

## 2. Proposed Properties of the NO/ONOO-Cycle

**Figure 1:**
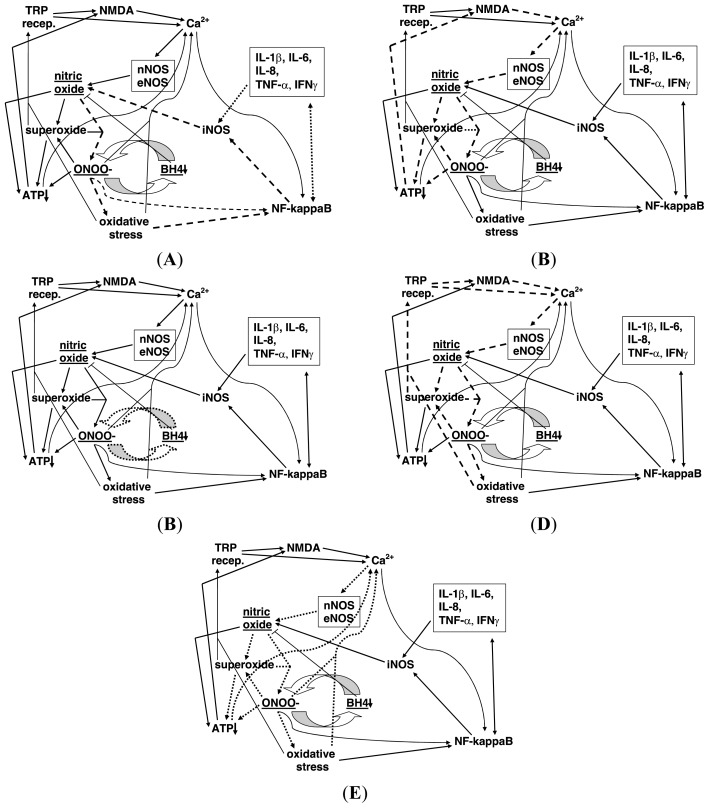
**A–E** are essentially identical diagrams of the proposed NO/ONOO-cycle; each arrow represents one or more mechanisms whereby one element of the cycle acts to increase the levels of a second element of the cycle (taken from ref. [1] with permission). **A**–**E** differ from one another in which arrows are dashed, so that the constituent cycles that make up the overall NO/ONOO-cycle may be considered independently of each other. Each of these five cycles is discussed in some detail in the text. Each of the five underlying cycles is expected to amplify the other underlying cycles through elevation of their common elements, generating a robust and difficult to down-regulate overall NO/ONOO-cycle. The 12 elements of the overall cycle are as follows: Nitric oxide (NO), superoxide, peroxynitrite (ONOO-), oxidative stress, NF-κB, four inflammatory cytokines and one chemokine (IL-8) in the upper right box, the inducible nitric oxide synthase (iNOS), mitochondrial dysfunction leading to lowered ATP generation, elevated cytosolic Ca^2+^, several of the transfer potential receptor (TRP) group of ion channels, the NMDA receptors, and tetrahydrobiopterin (BH4) depletion.

What is now known as the NO/ONOO-cycle has gone through several iterations [1–9], with the most recent version diagrammed in Figure 1A–E [1]. Subfigures A to E in Figure 1 are identical to each other, except that certain arrows in each are dashed, so that each of these, as discussed below, is a vicious cycle on its own. Each of the arrows in Figure 1 represents one or more mechanisms through which one element of the cycle can act to increase a second element. Near the core of the cycle (center, slightly left), nitric oxide (NO) reacts with another free radical, superoxide (^•^OO-) to form peroxynitrite (ONOO-), a potent nonradical oxidant, which is the most central element of the cycle.

Subfigures A to E in Figure 1 are separate, independent cycles which are predicted to interact with and amplify each other through elevation of their common elements. Thus, even in the absence of knowledge of the elements of the cycle, one can see that if these diagrams, Figure 1A–E, are correct, the combination of the five underlying cycles (Figure 1A–E) will through their interactions, form a robust and difficult to down-regulate compound cycle that we call the NO/ONOO-cycle. 34 specific mechanisms represented by these arrows are listed in the following section.

Let us start with the dashed arrows in Figure 1A starting with the reaction of NO with superoxide to form peroxynitrite (ONOO-). Elevated ONOO-, a potent oxidant, produces oxidative stress, an imbalance between oxidants and antioxidants. Both ONOO-and oxidative stress activate the transcription factor NF-κB (lower right) which activates, in turn the transcription of both the inducible nitric oxide synthase gene (iNOS) and also several inflammatory cytokines (box, upper right). Each of these cytokines is linked to NF-κB by a double headed arrow, such that each of them has its synthesis stimulated by NF-κB and most also, in turn, increase NF-κB activity and some of them can also increase iNOS induction independently of NF-κB. Some of the cytokines can also act independently of NF-κB to increase iNOS activity. Each of these activities, then, can produce increases in iNOS activity, leading in turn, to increased NO, thus producing a complete cycle.

There are also at least four other major cycles that are each parts of the overall NO/ONOO-cycle. The simplest of these is what is called the central couplet, the reciprocal elevation of ONOO-and depletion of tetrahydrobiopterin (BH4), (slightly below and right of center, Figure 1C) [9]. ONOO-is known to oxidize and therefore deplete BH4 and BH4 depletion is known to produce a partial uncoupling of the NO synthases (eNOS, nNOS and iNOS). When these NOSs are uncoupled, they produce superoxide in place of NO [9]. Because the reaction of these two compounds is extremely rapid, but there are mechanisms which lead to rapid loss or sequestration of them in the cell, the synthesis of both on nearby enzymes is expected to be particularly efficient in producing ONOO-, a potent oxidant. Thus ONOO-produces BH4 depletion which is expected to produce more ONOO-. This central couplet is thought to be particularly important in switching on the cycle [9], because NO acts to lower both NF-κB activity and NMDA activity, both important parts of the NO/ONOO-cycle. It may be argued, therefore that increasing the ratio of ONOO-to NO may be required to produce a chronic cycle and consequent chronic disease. This central couplet, as discussed below, may be particularly important to our understanding of HF, where BH4 oxidation and depletion and also partial NO synthase uncoupling have been shown to occur and where the ratio of ONOO-/NO appears to have additional important roles.

Other parts of the cycle (see Figure 1B) involve a very complex series of events, both intramitochondrial and largely extramitochondrial, leading to mitochondrial dysfunction and consequent ATP depletion (lower, left). The intramitochondrial sequence is often initiated by NO, but involves superoxide, ONOO-, inactivation of mitochondrial proteins and oxidation of the cardiolipin in the inner membrane in the mitochondrion. The extramitochondrial sequence is triggered by ONOO-, leading to major stimulation of poly(ADP-ribose) polymerase (often designated PARP or PARS) [1–3], leading to the depletion of the PARP enzyme substrate NAD and consequently also its reduced form, NADH. NADH is the most important source of reductants entering the mitochondrion and is essential, therefore for effective oxidative phosphorylation; NADH depletion, therefore, leads to mitochondrial dysfunction and ATP depletion. Lowered energy metabolism is known to act via two mechanisms to increase activity of the NMDA receptors [2,3,8] (Figure 1B, top center) which acts in turn to increase levels of intracellular calcium and consequent eNOS and nNOS activity (these both being calcium-dependent enzymes), leading to increased NO and ONOO-, feeding back into the mitochondrial cascade and ATP depletion.

An additional cycle (Figure 1D), includes five of the TRP group of receptors (upper left) which are known to be stimulated by oxidative stress, TRPA1, TRPV1, TRPC3 and TRPC5 and TRPM2 [2,10]; these and other members of this receptor group are also reported to be stimulated by NO. The NMDA receptors, glutamate receptors involved in producing excitotoxicity act, as do the TRP receptors to increase intracellular calcium levels, which act in turn, to stimulate two of the calcium dependent NOSs, eNOS and nNOS, leading back to increased NO, superoxide and ONOO-and oxidative stress, leading in turn to increased activity of some of these TRP receptors.

Figure 1E is focused on the properties of the plasma membrane calcium ATPase, which acts to pump excessive intracellular calcium out of the cell, an enzyme which is inactivated by both ONOOand other oxidants and, being an ATPase, its activity will be, of course, lowered by lowered energy metabolism [1]. All of these interact with each other (Figure 1E) to form another complex vicious cycle. In HF, calcium pumping and lowering of intracellular Ca^2+^ is performed primarily by the sarcoplasmic reticulum enzyme SERCA2a calcium-ATPase, which is also inactivated by peroxynitrite-mediated nitration and by oxidation, as discussed below.

Important, testable predictions of the overall NO/ONOO-cycle are discussed in the second section, below.

## 3. Thirty Four Specific NO/ONOO-Cycle Mechanisms

What has become known as the NO/ONOO-cycle has become increasingly complex over time, as it has become clear that additional mechanisms should be considered as integral parts of the cycle. The current list of cycle mechanisms is given below (taken from ref. [1], with permission). Documentation for each of these 34 is provided in ref. [1].

1. Extremely rapid, diffusion limited reaction between nitric oxide (NO^•^) with superoxide (OO^•−^), forming peroxynitrite (ONOO-).
2. ONOO-, a potent oxidant, can act to increase the activity of the transcription factor NF-κB.
3. ONOO- breaks down both before and after reaction with carbon dioxide into the following free radicals, hydroxyl (HO^•^), carbonate (CO_3_^•^) and NO_2_ radical (NO_2_^•^), each of which are responsible for a number of consequences produced by ONOO-.
4. ONOO- being a potent oxidant produces oxidative stress, an imbalance between oxidants and antioxidants.
5. Oxidative stress also produces increases in NF-κB activity because its activity is stimulated by oxidants and inhibited by chain-breaking antioxidants.
6. NF-κB produces increased transcription of the inducible nitric oxide synthase (iNOS), a gene whose transcription is known to be stimulated by NF-κB elevation and whose elevation also stimulates much of the inflammatory cascade.
7. NF-κB also stimulates the transcription of several inflammatory cytokines, including IL-1β, IL-6, IL-8, TNF-α, and IFNγ.
8. Each of the cytokines listed in 7 above, act directly and/or indirectly to stimulate the transcription of the iNOS gene, acting in some cytokines via the double headed arrow linking them to NF-κB and also, acting in some cytokines directly on iNOS induction.
9. When iNOS is induced, it produces large amounts of NO.
10. ONOO- inactivates the plasma membrane calcium-ATPase, leading to lowered calcium extrusion and increased levels of intracellular calcium.
11. Other oxidants inactivate the plasma membrane calcium-ATPase, leading to increased levels of intracellular calcium; such inactivation of the calcium ATPase has substantial pathophysiological effects and may well contribute to the prolonged impairment of calcium extrusion seen under circumstances where the NO/ONOO-cycle may have a role.
12. Lowered energy metabolism (decreased energy charge/ATP) also lowers calcium-ATPase activity, leading to increased levels of intracellular calcium, as predicted for such an ATPase.
13. While modest elevation of mitochondrial calcium, leads to increased ATP synthesis, substantial elevation of intracellular calcium leads to substantial increases in intramitochondrial calcium, leading to increased superoxide generation in the mitochondrion; large increases in mitochondrial calcium will lead, in some circumstances, to apoptotic cell death.
14. Intracellular calcium stimulates the nNOS and eNOS forms of nitric oxide synthase, both of which are calcium dependent enzymes.
15. Increased nNOS and eNOS activity both produce increased NO synthesis.
16. ONOO- oxidizes tetrahydrobiopterin (BH4), depleting BH4 levels.
17. BH4 depletion produces partial uncoupling of the three NO synthases, such that these enzymes, when uncoupled, produce superoxide in place of NO. Because of the very rapid reaction of these two compounds to produce ONOO-, this partial uncoupling involving nearby NOS enzymes is expected to produce an increase in ONOO- production.
18. Nicking of nuclear DNA by ONOO- and hydroxyl and other radicals can produce a massive stimulation of poly (ADP-ribose) polymerase (PARP) and consequent poly ADP ribosylation of chromosomal proteins, leading, in turn to a massive depletion of NAD/NADH pools, because NAD is the substrate for such poly ADP-ribosylation. NADH depletion lowers, in turn, ATP production in the mitochondrion.
19. Other changes causing ATP depletion come from a cascade of events occurring within the mitochondrion. The cascade starts with NO, possibly produced by mitochondrial NO synthase (mtNOS which is thought to be largely a form of nNOS), with NO binding to cytochrome oxidase, competitively inhibiting the ability of molecular oxygen to bind. This inhibits the ability of cytochrome oxidase to serve as the terminal oxidase of the mitochondrial electron transport chain.
20. The action of NO in 19 above, produces increased superoxide production by the electron transport chain.
21. ONOO- in the mitochondrion also acts to produce increased superoxide from the electron transport chain.
22. Peroxynitrite (ONOO-), superoxide and their products lead to lipid peroxidation of the cardiolipin in the inner membrane of the mitochondrion. Cardiolipin is highly susceptible to such peroxidation, because most of the fatty acids that make up its structure in mammals are polyunsaturated fatty acids, which are much more susceptible to peroxidation than are other fatty acids.
23. Cardiolipin peroxidation leads to lowered activity of some of the enzymes in the electron transport chain, leading to further lowering of ATP synthesis.
24. Cardiolipin peroxidation also leads to increased superoxide generation from the electron transport chain in the mitochondrion.
25. ONOO- produces inactivation of the mitochondrial superoxide dismutase (Mn-SOD) as well as the copper-zinc superoxide dismutase, leading in turn to increased superoxide levels.
26. ONOO-, superoxide and NO all inactivate or inhibit the aconitase enzyme, lowering citric acid cycle activity and subsequent ATP synthesis.
27. Oxidative stress leads to oxidation of cysteine residues in the enzyme xanthine reductase, converting it into xanthine oxidase which produces superoxide as a product, thus increasing superoxide generation.
28. Increased activity of the enzyme NADPH oxidase, which produces superoxide as a product, is an important part of the inflammatory cascade, and contributes, therefore, to the cascade by producing increased superoxide. (Note: Increased NADPH oxidase is produced through the action of angiotensin II in cardiovascular diseases, including HF).
29. Activation of the NMDA receptors, produced as described in 31 and 32, below, allows calcium influx into the cell, raising intracellular calcium levels including mitochondrial calcium levels.
30. Activity of transfer receptor potential (TRP) receptors also allows calcium influx into the cell, again raising intracellular calcium levels, presumably leading to increased nitric oxide production.
31. The main physiological agonist of the NMDA receptors is glutamate whose extracellular concentration is lowered after release, by energy dependent transport. It follows that ATP depletion produces increased NMDA stimulation by lowering glutamate transport.
32. The activity of the NMDA receptors is also greatly increased by ATP depletion within the cells containing these receptors. The mechanism here is that the ATP depletion produces partial depolarization of the plasma membrane, which produces, in turn, increased susceptibility of the NMDA receptors to stimulation.
33. Several of the TRP group of receptors have been shown to be stimulated by increased superoxide and/or oxidative stress or their downstream consequences, these being the TRPV1, TRPA1, TRPC3, TRPC5, TRPM2 and TRPM7 receptors, being produced in part through the oxidation of cysteine residue side chains. Several TRP receptors are also activated by nitric oxide mediated nitrosylation.
34. TRPV1, TRPA1 and probably several other TRP group receptors, receptor stimulation has each been repeatedly shown to lead to increased NMDA activity, with neurons containing these TRP family of receptors acting in part by releasing glutamate, the major physiological NMDA agonist.

We have, in summary, 34 distinct, well documented biochemical/physiological mechanisms that make up the complex vicious cycle we call the NO/ONOO-cycle. Most if not all of these are well-accepted biochemistry and physiology and most if not all of these 34 have been shown to play pathophysiological roles in one or more diseases. Consequently, there is little that is new regarding the cycle, except that when the individual mechanisms are put into juxtaposition with each other, they constitute a series of interacting cycles (Figure 1A–E) which based on their interactions, are likely constitute a robust vicious cycle, the NO/ONOO-cycle, which is likely to be a major challenge to effectively down-regulate.

## 4. Five Principles that Can Be Used to Test for NO/ONOO-Cycle Diseases

There are five principles that underlie the NO/ONOO-cycle [1–4], each of which makes predictions that can, therefore, be used to determine if a specific disease may be a probable NO/ONOO-cycle disease:

1. Stressors that initiate the disease are able to act by raising cycle elements.
2. The various elements of the cycle, with the possible exception of NO [9], should be elevated in the chronic phase of the disease.
3. The correlates (symptoms and signs) of the disease should be produced by one or more elements of the cycle.
4. The basic mechanism of the cycle is local and such that it is localized to different tissues in different individuals. The reason for this primarily local nature is that the three inorganic compounds involved, NO, superoxide and ONOO-, have limited half-lives in biological tissues. And the mechanisms of the cycle, those various arrows, act at the level of individual cells. This allows for great variations in tissue distribution from one patient to another, producing a huge spectrum of illness. The point here is not that there are no systemic changes—clearly antioxidant depletion, neuroendocrine and immune system changes, the actions of some inflammatory cytokines and BH4 depletion will be to some extent systemic. But rather this primarily local nature gives much inherent variation due to the varying tissue localization of the basic mechanism (see Chapter 4 in ref. [3]). A correlate of the primarily local nature of the cycle is that different NO/ONOO-cycle diseases will differ from one another in what tissue or tissues must be impacted by the cycle in order to be diagnosed as a specific cycle-caused disease.
5. The cycle is the central cause of the disease, so that treatment of the disease should involve using agents that lower various parts of the cycle. In other words, we should treat the cause of the disease, not the symptoms. Other types of evidence showing causal roles for elements of the cycle, such as genetic evidence, also support this principle.

## 5. Role of Elevated NO/ONOO-Cycle Elements in HF

Evidence that relates to principles 2, 3 and 5 in HF are discussed together here. This is because relevant HF studies are often relevant to two or all three of these principles. We will be asking here: Is each specific cycle element elevated in HF? Does it have a role in producing the correlates (symptoms and signs) of HF? Does it have a role in producing the overall disease?

### 5.1. Peroxynitrite (ONOO-)

Because peroxynitrite is referred to in most of the HF literature by its name, rather than its chemical structure (ONOO-), it will be referred to by name in the remainder to this paper.

3-Nitrotyrosine (3-NT) is a marker for peroxynitrite, one that is studied through studies of free 3-NT, through histological studies using a 3-NT-specific antibody, or in some cases where a specific protein is highly susceptible to such tyrosine residue nitration, through studies of such a specific protein. Studies of the peroxynitrite role in HF, using peroxynitrite decomposition catalysts, peroxynitrite scavengers and/or peroxynitrite donors are also discussed.

Pacher *et al*. [11] studied doxorubicin-induced HF in a mouse model, implicated an essential role for peroxynitrite by using a novel peroxynitrite decomposition catalyst, FP15. They showed that FP15 not only preserved most cardiac function, it also prevented other effects of doxorubicin, including elevation of 3-nitrotyrosine, elevation of matrix metalloproteinases (MMPs) produced by activation of inactive precursors, and elevated malondialdehyde, a marker of lipid peroxidation. Similar protection was also found from aminoguanidine, a specific iNOS inhibitor or from using an iNOS mouse knockout, suggesting that iNOS was the origin of most of the nitric oxide precursor of peroxynitrite. MMP activation is thought to have an important role in the tissue remodeling that occurs in HF. Okamoto *et al*. [12] had previously shown that activation of inactive precursors of MMPs was produced by peroxynitrite, by protein oxidation and subsequent protein *S*-glutathiolation and Pacher *et al*. [11] suggest that this mechanism may explain this part of their study.

Lokuta *et al*. [13] showed high levels 3-NT at specific residue in SERCA2a in HF, leading to greatly lowered calcium pump activity and lowered rate of cardiac relaxation. This shows local elevation of peroxynitrite, causal role in HF specifically in producing an important sign of HF, namely lowered calcium pump activity and consequent lowering of the rate of relaxation. SERCA2a activity is not only decreased by peroxynitrite elevation and consequent nitration in myocytes, but is also decreased by oxidation and sulfonylation, showing a role for oxidative stress [14].

Mihm *et al*. [15] showed that the creatine kinase, a critical enzyme in energy storage and utilization in the myocardium, is nitrated and inactivated by peroxynitrite in HF. They had previously shown inactivation of creatine kinase by peroxynitrite and lowered myofibrillar creatine kinase (MM-CK) enzymatic activity in HF. They also showed [15] high levels of protein tyrosine nitration in HF. Low concentrations of peroxynitrite *in vitro* inactivated MM-CK, with tyrosine nitration paralleling the inactivation. Anti-3-NT antibody immunoprecipitated much of the MM-CK protein from failing hearts [15]. They conclude [15] that “cardiac ONOO-(peroxynitrite) formation and perturbation of myofibrillar energetic controllers occur during experimental heart failure; MM-CK may be a critical cellular target in this setting”.

It is not unusual for transplanted hearts to undergo severe ischemia-reperfusion leading to HF, such that such HF following transplantation is considered to be a consequence of a severe ischemia-reperfusion. Lauzier *et al*. [16], studying rat heart reperfusion leading to HF, showed that the peroxynitrite decomposition catalyst, FeTPPS confers cardioprotection in isolated rat hearts, showing that peroxynitrite has an essential role in ischemia-reperfusion caused HF.

Eleuteri *et al*. [17] studied markers of oxidative/nitrosative stress and other markers in chronic HF patients. They found elevation of 3-NT levels, oxidative stress markers and inflammatory cytokines and also BH4 depletion. This provides evidence for peroxynitrite elevation, as well as for other important changes in HF.

Bonilla *et al*. [18] showed that SIN-1, a peroxynitrite donor, can produce certain changes in myocyte electrophysiology similar to those found in HF, including changes in action potentials and Ca^2+^ cycling, strongly suggesting a causal role of peroxynitrite in such changes.

The study of Ferdinandy *et al*. [19], showed that the cytokines TNF-α, IL-1β and INFγ, acted in combination to cause failure of isolated working rat hearts, accompanied by raises in superoxide and xanthine oxidase and NADPH oxidase superoxide generation, iNOS induction, peroxynitrite. They showed that peroxynitrite had a causal role in this model of HF, because HF could be blocked by the peroxynitrite decomposition catalyst FeTPPS.

Peroxynitrite may also have a role in the loss of responsiveness (tolerance) to isoproterenol and other β-adrenergic agents in the treatment of HF. Kohr *et al*. [20] showed that when mouse myocytes were exposed to both isoproterenol and to the peroxynitrite donor SIN-1, the SIN-1 exposure greatly lowered the ability to isoproterenol to produce either increased Ca^2+^ transients or myocyte shortening. This SIN-1 effect was blocked by the peroxynitrite decomposition catalyst FeTPPS. β-adrenergic agents are thought to act here, by raising cAMP levels, which then act to stimulate protein kinase A phosphorylation of the Ser16 residue of phospholamban. This effect of SIN-1 on isoproterenol responses was missing when they used myocytes from a phospholamban knockout mouse strain. In “wild type” mouse myocytes, SIN-1 was shown to greatly lower phosphorylation of the Ser16 residue of phospholamban. Kohr *et al*. [20] argue that the effect of peroxynitrite here is to raise the activity of a protein phosphatase that dephosphorylates the Ser16 residue. Protein phosphatase 2A has been shown to dephosphorylate the Ser16 residue of phospholamban and has been shown to be nitrated on Tyr284, leading to increased phosphatase catalytic subunit levels and activity [21]. It seems likely, therefore, that peroxynitrite here acts via tyrosine nitration of Tyr284 of protein phosphatase 2, leading through the pathway of action outlined in this paragraph and to loss of β-adrenergic responsiveness. Whether this is the sole or main mechanism of action in loss of responsiveness to β-adrenergic agents in HF patients or experimental animals remains to be determined.

Katori *et al*. [22] studied the effects of infused peroxynitrite into hearts of normal and HF dogs. The effects of such infusion were distinct from effects of peroxynitrite in isolated cardiomyocytes, probably due to the short half life of peroxynitrite. Such infusion led to larger increases in nitrate/nitrite levels in HF dogs than in normals, suggesting a regulatory response in HF, producing a substantial increase in NO and peroxynitrite. They [22] also showed that β-adrenergic agents can be directly oxidized by peroxynitrite, suggesting an additional mechanism for loss of β-adrenergic responsiveness from that discussed in the previous paragraph.

However, the influence of peroxynitrite in this process is not entirely negative in terms of its effect on protein kinase A activity. Peroxynitrite has also been shown to be able to change protein kinase A, such that some enzyme molecules become cAMP-independent [23]. A second, possible mechanism that might be helpful is of *S*-nitrosylation of cardiac proteins, leading to increased contractility [24]. Such *S*-nitrosylation is produced both by peroxynitrite [24] and by reaction of nitric oxide with thiyl groups, with such groups being generated from thiols by reaction with free radicals [25]. There is also a potentially helpful mechanism through the activation of SERAC2a activity by nitroxyl [26]; here, however, the physiological linkage to peroxynitrite is uncertain.

Daiber *et al*. [27] find that hydralazine, a fairly effective peroxynitrite scavenger, is a useful therapeutic agent in HF and attributed its effectiveness to its ability to lower peroxynitrite levels.

Lancel *et al*. [28], studying a rat model of endotoxin-induced HF, found elevation of protein bound 3-nitrotyrosine (3-NT), oxidative stress, NF-κB elevation and TNF-α elevation. All of these were lowered, as was myocardial dysfunction by using the peroxynitrite scavenger mercaptoethylguanidine or the peroxynitrite decomposition catalyst, FeTPPS, showing that peroxynitrite has a causal role in these responses [28].

Other studies implicating peroxynitrite in HF are discussed below. The studies reviewed in this section show that peroxynitrite can be elevated in HF and that such elevation may act to cause HF and to produce changes in SERCA2a activity, creatine kinase activity, increased nitration of tyrosine residues, increased oxidative stress, increased oxidation of β-adrenergic agents, decreased phospholamban phosphorylation and apparently increased MMPs, each of which have specific roles in HF. Decreased phospholamban phosphorylation may be responsible, in part, for lowered β-adrenergic responsiveness. Furthermore, peroxynitrite decomposition catalysts or scavengers appear to be effective therapeutic agents in HF. While a few effects of peroxynitrite may be protective, such as its effect of protein kinase A and on *S*-nitrosylation leading to increased contractility, overall peroxynitrite has an important causal role in HF and in producing specific causal changes in HF.

### 5.2. Oxidative Stress and Superoxide

Superoxide and oxidative stress are typically intertwined with each other, given the essential role of superoxide both as a precursor of peroxynitrite, a common cause of oxidative stress and as an essential precursor of Fenton chemistry, the other common chemical origin of oxidative stress. Both oxidative stress and superoxide in HF will be considered together here. Both of these appear to have roles in HF, as discussed above [11,12,14,16,17,19].

There are substantial numbers of studies where markers of oxidative stress have been shown to be elevated in either animals or humans with HF. These include studies where such markers as isoprostanes, 8-iso-PGF2α, malondialdehyde, thiobarbituric acid reacting substances (TBARS), 4-hydroxy-2-nonenal, protein carbonyls or pentane were elevated or where reduced thiols were lowered (see for example [11,29–35]). Two of these [29,30] showed a correlation between the extent of oxidative stress, as shown by markers, and severity of HF, suggesting but not proving a causal role. Superoxide is generated in increasing amounts in HF, by complex I in the mitochondrion [35].

However, an earlier review expressed skepticism about a possible causal role of oxidative stress in HF or the use of antioxidants as possible therapeutic agents [36]. Since that time, some perhaps more convincing studies have been published.

Redout *et al*. [37] showed that both NADPH oxidase and complex II of the electron transport chain in the mitochondrion, both major sources of superoxide, have roles in increased production of reactive oxygen species in HF. Three studies implicate superoxide as having a causal role. Konorev *et al*. [38] studied the roles of superoxide and other oxidants in doxorubicin-induced HF in the rat. The two cell-permeable compounds, MnTBAP, a superoxide dismutase (SOD) mimic and ebselen, a peroxynitrite scavenger and glutathione peroxidase mimic, were both able to protect from doxorubicin-induced HF. The first compound provides evidence for a superoxide causal role and the second for a role of other oxidants. Faulk *et al*. [39], implicate both mitochondrial superoxide and oxidative stress in doxorubicin-induced HF, as well as cytosolic Ca^2+^. Melov *et al*. [40], showed that mouse knockouts for their mitochondrial superoxide dismutase (MnSOD), die shortly after birth of dilated cardiomyopathy. MnSOD knockout mice can be rescued [40], however, with the same SOD mimic used in [38], MnTBAP. After such rescue, they still die at a fairly young age, but apparently of brain dysfunction, presumably caused by the inability of MnTBAP to cross the blood-brain barrier. This study shows that increased superoxide in the mitochondrion can cause a form of HF, and that lowering such mitochondrial superoxide with MnTBAP can rescue these mice from HF. Nojiri *et al*. [41], used a heart muscle-specific mitochondrial Mn-SOD knockout mutant, showing the mutant mice developed HF with cardiac mitochondrial dysfunction and lowered ATP levels, clearly showing that elevated mitochondrial superoxide can cause HF.

A study by Kubin *et al*. [42] implicates superoxide as having a causal role in HF. They showed that rat heart failure, initiated by high levels of endothelin-1 (ET-1) had high levels of dihydroethidium oxidation, a measure of superoxide elevation. They showed cardiac protection [42] by using the SOD mimic, MnTBAP, from an antioxidant or from an NADPH oxidase inhibitor, with the first and third of these implicating elevated superoxide as having a causal role in HF and the second implicating oxidative stress. Ghosh *et al*. [43] found that hyperthyroid-caused heart failure changes were accompanied by both oxidative stress and mitochondrial dysfunction. Many of the specific changes seen were normalized by two antioxidants, melatonin and vitamin E, suggesting that the changes are caused, at least in part, by oxidative stress [43].

Dai *et al*. [44] showed that mitochondrial oxidants, presumably derived from mitochondrial superoxide, produced large changes in protein levels in the mitochondrial proteome in HF. They showed in a pressure overload model of HF in the mouse, that diverse changes in the mitochondrial proteome produced along with HF, as well as the HF itself, could be largely attenuated with a transgenic catalase gene, where the catalase product is targeted to the mitochondrion [44]. Catalase targeted to the peroxisome, was largely inactive. Because catalase has a high specificity for hydrogen peroxide, this suggests that mitochondrial hydrogen peroxide, presumably derived from mitochondrial superoxide, can have substantial effects in producing HF as well changes in the mitochondrial proteome in HF.

Matsushima *et al*. [45] showed that overproduction of the mitochondrial antioxidant protein peroxiredoxin-3 protected from left ventricular remodeling, including both cavity dilatation and dysfunction, in a transgenic mouse study of HF. Peroxiredoxins are peroxide reductases and have been shown to act as peroxynitrite reductases [46,47], suggesting that overproduction here may act to lower mitochondrial peroxynitrite. This finding argues for a causal role of mitochondrial peroxides, probably including peroxynitrite, in causing certain symptoms and signs of HF. Peroxiredoxin-6 was shown to be strongly induced by cardiac-specific overexpression of iNOS [48], suggesting that this may be a protective regulatory response, lowering peroxynitrite.

Wang *et al*. [49] showed that mitochondrial oxidative stress in HF produces oxidative changes to enzymes required for mitochondrial ATP synthesis, producing mitochondrial energy metabolism dysfunction.

Tsutsui *et al*. [50] found that oxidative stress in the mitochondria of the myocardium, generated a series of changes that can be prevented or lowered by mitochondrial overexpression of the antioxidant protein peroxiredoxin-3. These included myocyte hypertrophy, apoptosis, interstitial fibrosis and MMP activation, producing maladaptive cardiac remodeling and failure, oxidative mtDNA damage and lowered mtDNA copy number.

A number of studies report oxidation of the mitochondrial cardiolipin in HF [51–53]. Because cardiolipin is a phospholipid in the inner membrane of the mitochondrion, containing in the heart four polyunsaturated linoleic acid residues which are highly susceptible to lipid peroxidation, high levels of oxidants in the mitochondrion in HF may be predicted to lead to increases in cardiolipin peroxidation in the myocardial mitochondria.

Tsutsui *et al*. [54] have written an extensive, informative review on how mitochondrial oxidative stress generated both mitochondrial dysfunction and myocardial remodeling in HF.

Finally, there are a number of studies on the role of low molecular weight antioxidants as HF preventive or protective agents [55–60], suggesting causal roles of oxidative stress in HF.

### 5.3. NF-κB

There are several studies showing NF-κB elevation in HF and that certain aspects of HF can be lowered by using an agent that lowers NF-κB activity.

Tanaka *et al*. [61] studied a pressure overload, thoracic aortic constriction murine model of HF. They showed that IMD-1041, an inhibitor of IκB kinase-β regulator of NF-κB, which lowers NF-κB activation, lowers several correlates of HF that are produced by such pressure overload. IMD-1041 lowered cardiac histological changes, including cardiac fibrosis and cardiomyocyte hypertrophy [61]. It also lowered the nuclear translocation of p65 and activity of MMP-2, increased fractional shortening and lowered cardiac fibrosis. They conclude [61] that lowering NF-κB activity may be useful in preventing pressure overload cardiac dysfunction.

Cai *et al*. [62] showed in a rat model of HF, that epigallocatechin-3-gallate (EGCG), a polyphenolic compound found in green tea that is known to lower NF-κB activity, acts to lower a number of HF associated changes, including elevated connective tissue growth factor levels, elevated collagen and fibronectin synthesis and cell proliferation of cardiac fibroblasts. They also showed that ECGC acts to lower the nuclear translocation of the NF-κB p65 subunit. Cai *et al*. [62] conclude that their findings are the first evidence that EGCG acts to lower cardiac fibrosis by lowering NF-κB activity.

Maier *et al*. [63] used a very different approach to show an NF-κB role. They constructed a double transgene mouse, where high level activity of NF-κB specific to the myocardium could be turned on by removing doxycycline from the drinking water, allowing them to look at the effects of such myocardial-specific high NF-κB activity [63]. They found that such high NF-κB activity in the myocardium produces myocarditis, inflammatory dilated cardiomyopathy and muscle fiber atrophy. Both ventricles and atria were dilated with strong systolic dysfunction and also some diastolic dysfunction [63]. There was also a substantial elevation of inflammatory cytokines and chemokines. They state that when the high NF-κB activity was turned off by restoring doxycycline to the drinking water, “the disease process was almost completely reversed.” Clearly this study shows a very substantial potential causal role for elevated NF-κB activity in many of the aspects of HF. The reversibility of most of the disease process studied is intriguing for the prospects of effective HF treatment.

Purcell *et al*. showed that NF-κB activity was implicated in producing cardiac hypertrophy [64], and Li *et al*. [65] showed that NF-κB activity was required for the hypertrophic response [65].

Xu *et al*. [66] implicate both elevated NF-κB and TNF-α activity in doxorubicin-mediated HF. Xing *et al*. [67], show that NF-κB activity is elevated in HF rats and that this elevation is responsible, as predicted by the NO/ONOO-cycle, for the elevation of the inflammatory cytokines TNF-α, IL-1β and IL-6. This leads us into the next issue, considered below, that of elevated inflammatory cytokines and other inflammatory markers in HF. A role for elevated NF-κB in HF is also supported by the study of Ock *et al*. [68], showing that raising NF-κB by lowering RANKL levels produces a substantial myocardial inflammatory effect, including raised levels of TNF-α and IL-1β. del Vescovo *et al*. demonstrated myocardial NF-κB induction of IL-6 [69].

Hamid *et al*. [70] found a strong increase in NF-κB activity in a mouse model of HF. When they compared a transgenic mouse strain with greatly lowered NF-κB activity with mice with normal NF-κB activation, they found major lowering in HF-related responses and greatly improved cardiac function is produced in the low NF-κB mouse strain. Specifically, they found the transgenic strain had improved survival and systolic function and lowered chamber remodeling, as well as lowered cytokine expression, fibrosis and apoptosis [70]. This study clearly shows that elevated NF-κB activity has a major causal role in this mouse model of HF.

### 5.4. Inflammatory Cytokines and Other Inflammatory Proteins

There are many studies showing that HF is associated with elevated levels of the cytokines TNF-α, IL-1β and IL-6 and of the inflammatory chemokine IL-8 and also of C-reactive protein. In some studies a number of other inflammatory markers have been studied in HF in addition to these and may be elevated as well. As noted in the previous section [67–69], NF-κB elevation causes much of the elevation of TNF-α, IL-1β and IL-6 in HF. There have also been quite a number of studies, showing that levels of these inflammatory regulators are correlated with severity of measures of HF dysfunction, but of course such correlations do not imply causality. Somewhat similarly, some agents that appear to be useful in the treatment of HF lower these cytokine levels, but again, because these agents affect other aspects of HF, not directly related to cytokine levels or action, this should not be interpreted as demonstrating causality of these cytokines in HF. Borthwick *et al*. [71] show that inflammatory cytokines can mediate tissue fibrosis, suggesting by not proving that they may have a role in such fibrosis in HF.

Finkel *et al*. [72] showed that three inflammatory cytokines, TNF-α, IL-6 and IL-2, all inhibited contractility of isolated hamster papillary muscles in a concentration-dependent, reversible manner. They showed that this lowered contractility was produced by NO, being inhibited by a competitive NO synthase inhibitor, l-NMMA, with such inhibition being prevented by high concentrations of l-arginine.

Kubota *et al*. [73,74] have shown, in a cardiac specific transgenic mouse model, that elevation of TNF-α levels can induce cardiomyopathy leading to HF. However, the levels of TNF-α produced here may be quite high, possibly well above those found in “normal” HF. Furthermore, studies with TNF-α receptor blockers have, in general, failed to produce a major improvement in HF, suggesting that this cytokine alone has a minor causal role in causing HF under most circumstances.

Yajima *et al*. [75] constructed a cardiac specific knockout of the *suppressor of cytokine signaling-3* (*SOCS3*) gene in the mouse. That gene knockout, which acts to increase cytokine signaling, produced increased cardiomyocyte mortality, contractile dysfunction and ventricular arrhythmia.

Perhaps the most convincing study suggesting that combinations of cytokines may have substantial causal roles in HF, is the study of Ferdinandy *et al*. [19], in which they studied the effects of IL-1β, IFNγ, and TNF-α in combination on isolated rat hearts. These cytokines produced a substantial decline in cardiac function, accompanied by raised levels of iNOS, NO, superoxide and peroxynitrite. Using specific agents, they were able to show that NO, superoxide and peroxynitrite each have causal roles in producing the cardiac function decline [19]. Perhaps the apparent much greater effectiveness of groups of cytokines in initiating HF may be due to their synergistic action, such as the well-documented synergism of IFNγ, and TNF-α in activating NF-κB [76].

IL-6 has been shown to enhance fetal gene expression and cardiomyocyte growth [69], suggesting a causal role for this cytokine in generating these signs of HF.

### 5.5. iNOS

Probably the first report of iNOS induction in the myocardium, was found by Schultz *et al*. [77] who showed that treatment of the rat heart myocytes with either endotoxin or a combination of TNF-α and IL-1β, induced the calcium-independent NO synthase we now know as iNOS.

Endothelin-1 initiation of HF is accompanied by large increases in iNOS activity, and Kalk *et al*., showed that a mouse iNOS knockout was largely protected from endothelin-1 initiated HF [78]. They concluded that endothelin-1 induced cardiac injury “is at least partially mediated by iNOS”.

Bacterial endotoxin exposure produces several types of cardiovascular dysfunction, including HF. There are several studies showing a large induction of iNOS in endotoxin-induced HF and also a large increase in NO levels [79–82]. In these studies, endotoxin produces both cardiac dysfunction and also a number of specific correlates of HF. iNOS appears to have a causal role in producing these HF responses, because a specific iNOS inhibitor, aminoguanidine, greatly lowers these effects [80,81]. The role of other NO/ONOO-cycle elements in bacterial endotoxin HF is discussed below in the section of various NO/ONOO-cycle elements in HF initiated by various stressors.

Schrader and colleagues [48,83] have developed a transgenic mouse, cardiac-specific overexpression model of HF, in which iNOS overexpression in the myocardium causes HF. The level of iNOS overexpression is high, so that the level of iNOS expression found in “normal” HF may not be the predominant causal element under most circumstances. Such iNOS overexpression was found to produce an up-regulation of peroxiredoxins in the heart [48], possibly as a protective mechanism to protect from peroxynitrite elevation. The protective activity of cardiac myoglobin in this model [83], strongly suggests that NO has a causal role, because of the action of myoglobin in scavenging NO.

Mungrue *et al*. [84] developed another transgenic mouse model with cardiac specific iNOS overexpression, where the *iNOS* transgene was also regulated by tetracycline. *iNOS* transgene expression produced mild inflammatory cell infiltrate, cardiac fibrosis, hypertrophy, and dilatation, leading to infrequent HF but common sudden cardiac death due to bradyarrhythmia. In this model, then, iNOS overexpression in the heart is sufficient to cause sudden cardiac death due to bradyarrhythmia but only infrequent HF. Perhaps differences in pattern or level of expression may explain the differences between this model and the one described in the previous paragraph.

Drexler *et al*. [85] performed a study on the action of iNOS in failing human hearts, obtained after transplant, comparing them with normal hearts that could not be used for transplantation because of no suitable available recipient. This was in response to earlier studies showing that “Heart failure is associated with activation of cytokines and expression of inducible nitric oxide synthase…”. They showed that iNOS mRNA and activity levels were elevated in failing heart tissue and that the iNOS level correlated with lowered response activity of these hearts to the β-adrenergic agent isoproterenol. They further demonstrated, confirming earlier studies, that NO was causing this lowered responsiveness, using a NO synthase inhibitor and two NO donors.

Haywood *et al*. [86] compared hearts from patients with three different types of HF with those of controls, finding iNOS expression in most of the HF hearts but in none in control hearts. They concluded that “iNOS expression occurs in failing human cardiac myocytes and may be involved in the pathophysiology of dilated cardiomyopathy, ischemic heart disease and in valvular heart disease”.

In a mouse model of hypertension, that mimics long-term hypertension in humans, Dias *et al*. [87] showed that consequent cardiac contractile dysfunction was long delayed in an iNOS knockout.

It can be seen from the above, that iNOS levels are elevated in failing hearts, possibly generated by elevated cytokine levels and NF-κB levels, and that it acts in animal models of HF both as a cause of HF and of specific symptoms and signs of HF. Several of these studies also document a role of elevated NO in HF.

### 5.6. NO

It have often been stated that lowered NO bioavailability has a role in causing HF. We have already seen studies in the previous sections strongly suggesting that this is not correct, because iNOS activity is elevated in HF and iNOS, NO and peroxynitrite each contribute to the development of HF. Damy *et al*. have shown that nNOS protein levels and activity are elevated in human failing hearts [88], although it is well established that eNOS levels are lower in HF.

This section starts out by asking whether HF leads to higher or lower NO synthesis. Most of the studies that have investigated this question have used nitrate/nitrite as a marker of NO, measuring what are essentially whole body levels in the blood. Winlaw *et al*. [89] found that plasma levels of nitrate in HF patients were approximately double those of healthy controls. Sugamori *et al*. [90] comparing HF patients with healthy controls, found that nitrate/nitrite levels were elevated in HF, as were TNF-α levels and that within the HF group, nitrate/nitrite levels were positively correlated with both TNF-α and HF severity. Usui *et al*. [91] found that nitrate/nitrite levels, TNF-α levels and levels of asymmetric dimethylarginine (ADMA, the endogenous NO synthase inhibitor) were elevated in HF patients *vs*. normal controls; within the HF group, nitrate/nitrite was positively correlated with ADMA levels and negatively correlated with ejection fraction. ADMA is regulated through regulation of dimethylaminohydrolase (DDAH) and has been reported to be elevated in a number of studies of HF. It is interesting, therefore, that ADMA was positively correlated with nitrate/nitrite in this study [91] and that, in general, these NO markers are elevated in HF despite ADMA elevation.

In a study of spontaneously occurring chronic valvular disease and dilated cardiomyopathy, de Laforcade *et al*. [92] measured nitrate/nitrite as a marker of NO and found it to be significantly elevated in diseased dogs compared with normal controls and also found it to be inversely correlated with fractional shortening. In another study of HF patients using nitrate/nitrite as a measure of NO, Yu *et al*. [93] found that NO levels were significantly elevated in both diastolic HF (ejection fraction >50%) and in systolic HF (ejection fraction <50%) compared with normal controls, with higher NO in systolic HF; both patients on oral organic nitrate drugs and those who were not were elevated *vs*. controls, however, showed higher levels among those on such drugs. In patients with left ventricular systolic failure, NO levels correlated with severity.

While the above-discussed studies all support the view that NO production in the failing heart is likely to be elevated, compared with controls, there are also two studies that have questioned this interpretation.

One is the study in dogs by Bernstein *et al*. [94], who found that nitrate levels in blood were elevated in dogs with HF. However, they also found that the elevated levels were correlated with elevated levels of creatinine, a marker of renal dysfunction. They suggest therefore that the elevated nitrate/nitrite levels may be caused by lowered renal function, not by elevated NO synthesis in the myocardium in HF. This issue will be further considered later in this section.

A second study questioning this interpretation is that of Kaye *et al*. [95] who found that nitrate/nitrite levels in human HF patients, used as a marker for NO, were elevated in patients *vs*. healthy controls. However, when they measured this same marker, comparing the arterial levels with those in the coronary sinus, they found that the levels were lower in the coronary sinus, in other words Δ < 0 and that there was no significant difference between the Δ for the patients and those for the healthy controls [95]. They interpreted this to show that there was extraction of NO (or at least nitrate/nitrite) in the heart and that the NO synthesis in the heart was not different between the HF patients and controls. It is the author’s view that this study is flawed based on the use of nitrate/nitrite as a marker for NO under their experimental protocol. Under most conditions, this is a useful NO marker, but the measurement of changes in NO by using this marker in blood flowing into and out of the heart, is questionable, in my view. NO is unstable in most biological tissues, getting converted to nitrate/nitrite within a second or so. However, NO can bind rapidly to deoxyhemoglobin, forming fairly stable adduct, nitrosyl hemoglobin (NH), and the conditions in the heart are ideal for NO to rapidly react, in this way. The heart has the highest level of oxidative metabolism in the body and for this reason is highly perfused, such that much of the erythrocyte hemoglobin is both rapidly deoxygenated and is highly available for reaction with NO. It is the author’s opinion, that this study should have used the paramagnetic resonance assay for NH [96,97] which has been reported to be elevated in HF [98], to measure NO production in the heart, rather than the nitrate/nitrite assay, measuring the Δ of NH between the arteries and the coronary sinus. In the absence of that type of data, it is reasonable to infer as others have done in other studies, that the elevated blood levels of nitrate/nitrite found in this study of HF [95] indicates elevated NO production in the failing heart.

There are some studies where HF patients have been shown to have lowered NO synthesis, in the vasculature, leading to increased vascular tone and hypertension [99,100]. These may be due to raised systemic ADMA levels, to lowered systemic BH4 levels (both produced by HF) or both. In any case, lowered vascular NO production suggests that the rise in whole body markers of NO (nitrate/nitrite) may substantially underestimate the level of NO synthesis in the HF myocardium.

In summary, then, there are seven studies all showing elevated blood levels of nitrate/nitrite in HF. How these should all be interpreted is discussed in some detail later in this section.

Otani reviewed roles of NO in HF and more generally in myocardial repair and remodeling [101]. In that review, Otani contrasts “the identification of NO as the endothelium-derived relaxing factor combined with the discovery of NO generation by nitric oxide synthases…”, with “It is now apparent that NO and cognate reactive nitrogen intermediates are involved in a wide variety of *pathophysiological processes* in the cardiovascular system where it orchestrates a plethora of cellular activities in cardiomyocytes…” (italics added). Most of the physiological effects of NO are mediated through its ability to stimulate the soluble guanylate cyclase (sGC), producing cGMP which acts, in turn to stimulate the cGMP-dependent protein kinase (protein kinase G) [102,103] or through its ability to nitrosylate protein cysteine residues. In contrast to that, most of the pathophysiological effects are through the reaction of NO with superoxide to produce peroxynitrite [104,105]. There are some exceptions to that pattern that Otani [101] discusses, but that is the general pattern.

Let us look how these two pathways of action may interact with each other in the context of HF and other cardiovascular diseases.

The activity of the NO/sGC/cGMP/protein kinase G pathway responses can be assessed independently of NO reacting with superoxide to form peroxynitrite, by using agents that increase the enzymatic activity of sGC, called stimulators or activators [106,107] (these are distinct from one another, as discussed below [108,109]) or the use of sildenafil [110,111], a phosphodiesterase-5 (PDE5) inhibitor, which produces lowered hydrolysis of cGMP and therefore raises cGMP levels. Each of these studies showed that activation of this pathway leading to protein kinase G activation, lowers cardiac hypertrophy and improves overall survival [108–111], with improved systolic and diastolic function, lowered cardiac remodeling, lowered inflammation, and lowered apoptosis also being reported [110,111]. Most importantly, activating this pathway also lowers superoxide generation acting in part, by lowering NADPH oxidase [108,109], a major source of superoxide in HF. In this way, then activating the sGC/cGMP/protein kinase G pathway, lowers both HF and multiple correlates of HF, acting, in part, via lowered superoxide. The lowered superoxide will be expected to lower the reaction of NO with superoxide to form peroxynitrite.

However, the converse is also true, the reaction of NO with superoxide to form peroxynitrite and consequent oxidative stress is likely to greatly lower the NO/sGC/cGMP/protein kinase G pathway. One mechanism leading to this conclusion is that the sGC, is a heme enzyme, where NO activates the enzyme by binding to the heme group, binding specifically to Fe II iron of the heme. Synthetic stimulators of this enzyme, mentioned in the previous paragraph, also bind and increase the activity of this same heme-containing enzyme [106,107]. However, under oxidative stress, oxidants can convert the sGC Fe II iron to Fe III, which is an unstable form that can lead to release of the porphyrin from the apoenzyme. Both the Fe III form and the apoenzyme form of sGC are activated by “activators” but the Fe II form is not. It is of interest, then, that in animal models of HF, activators produce a better clinical response than do stimulators [106,108], suggesting that there is a lot of this oxidation occurring in these models of HF, thus limiting NO signaling through this pathway and consistent with a substantial role of oxidative stress in HF.

Another mechanism of cGC oxidative inactivation has been demonstrated by Crassous *et al*. [112], who have shown that *S*-nitrosylation of a specific cysteine residue in sGC blocks the enzyme from NO stimulation. Such *S*-nitrosylation has been shown to be produced by the reaction of NO with thiyl radicals [25]. Because thiyl radicals are produced by the reaction of free radicals with thiols, increased *S*-nitrosation may be expected to be produced by elevated peroxynitrite and consequent nitrosative stress, as reported by Crassous *et al*. [112]. BH4, acting as an antioxidant, has been shown to protect sCG from oxidative inactivation [113]; consequently the BH4 deficiency that occurs in HF (discussed below) may well contribute to increased sGC oxidation. *S*-Nitrosylation of sGC may be predicted to occur as a consequence of oxidative stress and iNOS induction in HF but has not been studied in HF, to my knowledge.

There are two additional pathways of action by which peroxynitrite/oxidative stress can act to lower signaling via the cGMP/protein kinase G. Protein kinase G has been shown undergo peroxynitrite-mediated high level tyrosine nitration on specific residue, leading to inactivation of the enzyme [114–117]. This has been most studied in pulmonary hypertension and consequently, there is no substantial evidence on whether or to what extent this occurs in HF. However, given that the evidence, discussed above, for other roles of protein tyrosine nitration in HF, it seems plausible and perhaps even likely that this mechanism may produce a substantial lowering cGMP/protein kinase G signaling in HF.

The studies of Liu *et al*. [118], suggest that protein relocalization as a consequence of remodeling in HF, may also negatively influence sGC/cGMP/protein kinase G signaling although the physiological effects on such signaling must be viewed as speculative. Finally, there is a fourth mechanism, predicted to influence this pathway. Lu *et al*. [119] showed that oxidative stress increases expression of PDE5, the phosphodiesterase that specifically hydrolyzes cGMP, in HF. Such increased expression of PDE 5 is expected to lower cGMP levels in HF and therefore lower the signaling of this pathway.

These changes, produced under the influence of peroxynitrite/oxidative stress, that lower the activity of the NO/sGC/cGMP/protein kinase G pathway may appear to be caused by lowered NO bioavailability, as has been widely argued. However, they are much more easily interpreted in terms of lowered activity of subsequent steps in that pathway. Only two of the four mechanisms, oxidation of Fe II heme in sGC to FE III and increased PDE5 expression produced by oxidative stress have been studied and shown to occur in HF. The other two, nitrosylation of sGC and tyrosine nitration of protein kinase G have been shown to occur in other cardiovascular disease contexts but may be predicted to occur in HF, due to elevation of both peroxynitrite and oxidative stress. However, whether two, three of all four of these occur in HF, they suggest that the data purportedly arguing for lowered NO bioavailability in HF have been misinterpreted. The bulk of the evidence on NO synthesis discussed in this section argues for its levels being high rather than low. The evidence discussed in the previous sections argues still more compellingly that NO levels in HF are high, rather than low and that high levels of iNOS, NO and peroxynitrite each have important roles in the etiology of HF.

Lowered cGMP synthesis and protein kinase G enzymatic activity may be expected to not only substantially lower NO signaling through this pathway in HF, they also lower protective signaling produced by brain natriuretic peptide (BNP) and atrial natriuretic peptide (ANP). Both BNP and ANP act to protect the myocardium from HF, by acting along the cGMP/protein kinase G pathway [120–122]. However, the form of guanylate cyclase that is stimulated by BNP and ANP is the membrane-bound enzyme, not by sGC [122]. The study of Takahashi *et al*. [120] suggests that there is greater dysfunction in cGMP response to ANP/BNP in serious HF (NYHA IV) than for the cGMP response to NO. While such signaling via increased cGMP by either NO or BNP/ANP is not completely missing in the failing heart [120], it appears that signaling via the plasma membrane guanylate cyclase in HF may be lowered more than is signaling via sGC.

The role of NO in HF is addressed by the Ferdinandy *et al*. study of cytokine-induced HF in isolated working rat hearts [19], discussed above. They showed that the NO elevation was causal because HF could be blocked using an NO synthase inhibitor. Furthermore, because superoxide and peroxynitrite levels were elevated as well and peroxynitrite was shown by using a peroxynitrite decomposition catalyst to be causal [19], their results strongly suggest, as they inferred, that NO was acting in HF induction by acting as a peroxynitrite precursor.

An interesting study on these two pathways of NO action was published by Arstall *et al*. [123], studying the induction of apoptosis in cardiac myocytes by the two cytokines, IL-1β and IFNγ, acting together. They showed that apoptosis was induced not only by these two cytokines but also be an NO donor and by peroxynitrite. Furthermore cytokine-induced apoptosis was blocked by two NO synthase inhibitors, one nonspecific and the other iNOS specific and by the peroxynitrite decomposition catalyst, MnTBAP, showing that both NO and peroxynitrite have a causal role in producing cytokine-induced apoptosis. In contrast, the NO/sGC/cGMP/protein kinase G pathway had no apparent role, because apoptosis was not lowered by inhibition of sGC nor significantly influenced by the hyperstable cGMP analog, 8-bromo-cGMP. They complete their paper [123] by stating “It appears possible that if substantial expression of iNOS occurs in cardiac failure, the actions of ONOO-generated on mechanical function, energetics and on pathways leading to death of cardiac myocytes, may all be contributing factors to its pathophysiology and progression.”

In conclusion, there is no evidence that the common statement about HF, that HF is caused in part by lowered NO bioavailability, is correct. It is the case that such NO bioavailability is limited in HF by lowered eNOS activity, by BH4 depletion (discussed below) and by accumulation of asymmetric dimethyl-arginine (ADMA). However, this seems to be more than compensated by a large increase in iNOS (discussed in the previous section) and possibly also by increased nNOS activity and Ca^2+^ stimulation of nNOS and eNOS. Consequently, NO levels are high rather than low. NO has an important role in causation of HF and correlates of HF, acting through peroxynitrite and potential NO-mediated protection by its action through the sGC/protein kinase G pathway may be largely inactivated two mechanisms of sGC oxidation, induction of PDE5 and protein kinase G nitration.

### 5.7. Mitochondrial Dysfunction (ATP Depletion)

Mitochondrial dysfunction in HF has been documented in many reviews, including lowered oxidative phosphorylation, glycolytic activity and consequent lowering of ATP synthesis [124–132]. Linkage of mitochondrial energy metabolism dysfunction in HF to the mitochondrial oxidative stress, has also been made [54,124,126,130,131] as has linkage to Ca^2+^ mitochondrial overload [133]. A number of reviews have argued for the use of therapies improving mitochondrial function for the treatment of HF [124,125,131,132].

Some of the best evidence supporting a causal role of mitochondrial dysfunction in HF, comes from three studies using transgenic mice carrying cardiac-specific mutations in genes each thought to have a specific role in mitochondrial function. Two of these studies showed that deletion or functional disruption of a gene with a specific role in mitochondrial fusion causes HF [134,135] and systolic dysfunction [135]. Cardiac-specific deletion of the *CRIF1* gene, a gene that has an important role in the insertion of oxidative phosphorylation proteins into the mitochondrial membrane, produces HF and also major changes in mitochondrial structure and lowered ATP production in the myocardium [136].

l-carnitine and its derivatives, which are thought to act by improving mitochondrial function, appear to be useful in treatment of HF, suggesting a causal role for mitochondrial dysfunction in HF [137–139]. Coenzyme Q10 (ubiquinone), which is also thought to act primarily in the mitochondrion, may also be useful in HF treatment, although this is still a controversial issue [140,141].

### 5.8. BH4 Depletion

There have been a series of studies, showing that BH4 depletion occurs in HF, as predicted by the NO/ONOO-cycle and that this leads to partial uncoupling of the nitric oxide synthases. For example, Silberman *et al*. [142], in a study of diastolic heart failure caused by hypertension in the mouse, showed that consequent lowering of BH4 levels produced partial uncoupling of the cardiac NO synthase activity and reduced NO synthesis, raised oxidative stress and superoxide production and increased levels of oxidized biopterins, all consistent with predictions of the cycle. Supplementing BH4, raised BH4 levels, partially normalized diastolic relaxation and phosphorylated phospholamban levels, showing that BH4 depletion has a causal role in producing these correlates of HF.

Takimoto *et al*. showed in a cardiac pressure overload mouse model of HF, that tissue remodeling and a series of changes clearly implicated in remodeling, fibrosis, myocyte hypertrophy, fetal gene expression and MMP-2/MMP-9 expression all increased, as did two measures of oxidative stress and 3-nitrotyrosine [143]. All of these decreased as did cardiac dysfunction when an otherwise isogenic nNOS knockout was used instead of the “wild type” mouse, showing that nNOS activity had a causal role in generating both tissue remodeling and and cardiac dysfunction and each of the additional changes, most of which are implicated in tissue remodeling. However, they also found that when BH4 was provided to “wild type” mice, the tissue remodeling disappeared and was replaced by concentrated nondilated hypertrophy [143]. The BH4 finding clearly shows that BH4 depletion is essential for producing the tissue remodeling and subsequent cardiac dysfunction and suggests that peroxynitrite, rather than NO itself may be the causal factor in producing these various changes.

Moens *et al*. [144] showed that lowered BH4 levels occur in a mouse model of HF induced by hypertension, linked to elevated oxidation of BH4 to dihydrobiopterin (BH2). They also showed [144] that supplementation with BH4 (via the drug sapropterin) was effective in treatment to lower both the partial uncoupling of the NO synthase activity and the consequent maladaptive tissue remodeling. Moens *et al*. [144] found that only a relatively narrow window of BH4 supplementation level was effective in such treatment, a window producing an increased BH4/BH2 ratio. This study shows that there is much oxidation of BH4 in HF, which the NO/ONOO-cycle predicts is caused by ONOOelevation, and that such oxidation and lowered BH4 levels produces both NO synthase partial uncoupling and maladaptive remodeling. Author’s note: There is large literature showing that raising reduced folates can lower NO synthase uncoupling, acting at least in part, by raising BH4 levels, suggesting that such folates may be useful in increasing the effectiveness of sapropterin in treatment of HF.

A number of other studies have demonstrated roles of BH4 oxidation and depletion in HF. Jessup *et al*. [145] showed that ovariectomy in the rat, can produce HF, with BH4 oxidation and depletion having an apparent role. They showed that L-VNIO, a partially specific NO synthase inhibitor, lowered superoxide production, limited remodeling and improved diastolic function.

Nishijima *et al*. ([146–148]) showed that BH4 depletion and oxidation and iNOS (NOS2) induction have roles in generating atrial fibrillation, a common correlate of HF, with elevated superoxide having an important apparent role. Treatment with BH4 and arginine leads to lowered atrial fibrillation. They conclude that “Modulation of NOS2 activity by repletion of BH4 may be a safe and effective approach to reduce the frequency of atrial arrhythmias during heart failure”.

Alkaitis and Crabtree [149] report that heart failure involves peroxynitrite oxidation of BH4, leading to NO synthase uncoupling, elevated superoxide production and BH2 elevation. They argue that BH4 depletion and NO synthase uncoupling “contribute to overload-induced heart failure, hypertension, ischemia/reperfusion injury and atrial fibrillation.”

Adiponectin [150] is “an anti-inflammatory, antiatherogenic adipokine elevated in heart failure (HF) that may protect against endothelial dysfunction by influencing underlying nitric oxide bioavailability.” Because such bioavailability requires BH4, this suggests that adiponectin may act by raising BH4 levels. Adiponectin has been shown to raise HSP90 levels [151,152] which acts to stabilize, in turn, both the eNOS form of NO synthase and also GTP cyclohydrolase I [153], the rate limiting step in the *de novo* biosynthesis of BH4. Interestingly, high levels of BH4 can also act to raise adiponectin levels [154].

While it seems likely that oxidation of BH4 by peroxynitrite is the primary mechanism for its depletion, another mechanism is suggested by the recent studies of Sharma *et al*. [155]. They showed that mitochondrial dysfunction leads to increased proteasomal degradation of the rate limiting enzyme in the biosynthetic pathway for BH4, GTP cyclohydrolase I, so it is possible that the mitochondrial dysfunction may have a role in lowering BH4 levels as well.

In general, then, the above discussed studies show that BH4 oxidation to BH2 occurs in HF, with such oxidation apparently being produced, at least in part, by peroxynitrite elevation. Such oxidation produces NO synthase partial uncoupling, leading to increased superoxide generation. This pathway apparently has a causal role in producing the following changes found in HF: tissue remodeling, abnormal phospholamban phosphorylation, diastolic dysfunction and atrial fibrillation. BH4 levels may also be important in HF through its interaction with adiponectin.

### 5.9. Cytosolic Calcium

Most cell types maintain low levels of cytosolic Ca^2+^, except when elevated Ca^2+^ levels are needed to produce regulatory responses. Clearly, myocyte Ca^2+^ level control is quite different from that, suggesting that if a Ca^2+^ elevation has a role in NO/ONOO-cycle elevation in HF, its role is likely to be distinct from that in most other tissues. In normal myocytes, cytosolic Ca^2+^ elevation is produced by ryanodine receptor-2 (RyR2) release of Ca^2+^ from the sarcoplasmic reticulum following Ca^2+^ influx through the plasma membranes via L-type voltage gated calcium channels, producing cardiac muscle contraction. This is followed by pumping of Ca^2+^ from the cytosol into the sarcoplasmic reticulum by the SERCA2a calcium pump mechanism and, to a lesser extent, Ca^2+^ pumping out of the cell by the plasma membrane calcium-dependent ATPase, producing cardiac muscle relaxation.

In HF, the Ca^2+^ raised transient is both reduced in amplitude and prolonged in duration, with the lowest Ca^2+^ levels being elevated [156,157]. These changes are thought to be produced primarily by leakiness of the RyR2 channels and lowered activity of SERCA2a [158]. Both of these contribute to lowered Ca^2+^ in the sarcoplasmic reticulum and both may also contribute to the changes in cytosolic Ca^2+^ transients [158]. SERCA2a lowered activity, as discussed above, is produced in part via peroxynitrite-mediated tyrosine residue nitration, which inactivates SERCA2a [13,159,160]. RyR2 leakiness is thought to be caused, in part by protein oxidation [161,162]. RyR2 proteins are also susceptible to tyrosine nitration, but whether it has a causal role in producing leakiness is uncertain [163]. Both RyR2 and SERCA2a activities are also strongly influenced by protein kinase A dependent phosphorylation [158]. Both SERC2a dysfunction and RyR2 leakiness are thought to have substantial causal roles in HF [158,164–166].

The complex differences in Ca^2+^ levels between normal and failing hearts, makes it difficult to determine whether elevated levels of Ca^2+^ may have a causal role in HF. Consequently, our approach here is to look at three important receptors for Ca^2+^ to determine whether elevated activity of these receptors have roles in HF. These receptors, calpain(s) a group of calcium-dependent cysteine proteases, calcium/calmodulin protein kinase II (CaMKII) and calcineurin, a calcium-dependent phosphoserine/phosphothreonine protein phosphatase, have each been shown to have roles in HF (Table 1). Calcineurin is known to act via activation of a pathway known as NF-AT or NFAT. Many of these studies involve the use of specific inhibitors that block the action of one of these three receptors. However, the calpain inhibitors typically inhibit all forms of calpain, and for this reason, calpain studies often leave open what form of calpain may be involved.

It can be seen from Table 1, that a wide variety of studies have shown roles of each of these Ca^2+^ receptors in generating various symptoms and signs of HF, as well as HF itself, allowing one to infer that Ca^2+^ has roles as well.

### 5.10. NMDA

While most studies of NMDA receptor activities have focused on roles of these receptors in the central nervous system, functional NMDA receptors have been found outside of neurons in many peripheral tissues including the myocardium. Huang and Su [186], following some previous studies of effects of NMDA antagonists on cardiac function in rats and pigs, studied the effects of the NMDA antagonist MK-801 on isolated rat cardiac tissues. They showed that MK-801 has a positive inotropic action in such rat cardiac tissue, suggesting that NMDA receptor activity has negative inotropic effects.

Matsuoka *et al*. [187] showed that the NMDA receptor blocker dizocilpine prevented sudden death in response to cold stress in cardiomyopathic hamsters, suggesting a role on of NMDA receptors in producing such sudden cardiomyopathic death. In this study, because the receptor blocker may be acting on all such receptors in the body, it is possible that action on NMDA receptors in the brain or elsewhere in the body, as opposed to in the myocardium, may be involved.

The most extensive studies of the role of NMDA-R1 receptors in the heart, specifically in HF have been published by Tyagi and colleagues in studies of the effects of hyperhomocysteinemia, which acts, in turn to increase susceptibility of HF. Homocysteine is a known NMDA agonist, but it can also act in other ways to produce possible pathophysiological effects. They showed that for a number of effects on cardiomyocytes thought to have roles in HF, homocysteine was acting as an NMDA agonist [188,189]. This was based on the finding that MK-801, an NMDA antagonist blocked responses to homocysteine [188] and that a cardiac-specific knockout of the R1 subunit of the NMDA receptor blocked cardiac responses to homocysteine in the mouse [189]. NMDA receptor-dependent responses to homocysteine included raised levels of MMP-9, which is thought to act in the mitochondrion to trigger the permeability transition involved in apoptosis, as well as other HF-related changes [188]. NMDA-dependent homocysteine responses also produced raised levels of NO [189], cytosolic Ca^2+^ and activity of the calcium-dependent protease, calpain [190]. Homocysteine also acts as an NMDA agonist to produce cardiac arrhythmia [190,191]. It follows that homocysteine acts in producing HF-related dysfunction by elevating one element of the cycle, excessive NMDA activity, which acts, in turn to cause elevation of other elements of the cycle as well as specific signs and symptoms of HF.

Two studies showed that stimulation of the NMDA receptors in cardiomyocytes leads to increased oxidative stress, mitochondrial dysfunction, NO, and inflammatory cytokines, leading to apoptosis [192,193]. Because the main direct effect of excessive NMDA activity is to open channels that allow Ca^2+^ to flow into the cell, the role of such excessive activity supports a role for excessive Ca^2+^ in HF.

Another study implicating excessive NMDA activity in HF, is the finding that domoic acid exposure appears to cause HF in sea otters [194]. Domoic acid acts both indirectly, through its stimulation of AMPA/kainate receptors, and directly to produce increased NMDA activity.

### 5.11. TRP Receptors

Three reviews of the role of the TRP receptors in HF [195–197] have been published, two of which [195,197] focus mostly on the TRPC group of receptors, particularly TRPC6 and TRPC3, but also including TRPC1 and TRPC4, all of which have substantial causal roles in the mouse model HF. That model allows for the study of transgenic mice and both cardiac specific gene knockouts and cardiac specific expression of dominant negative mutants have been used to clearly demonstrate causal roles of TRPC1, TRPC3, TRPC4 and TRPC6 in HF. TRPC3 and TRPC6 are known to be stimulated by diacylglycerol (DAG) whose production is increased through the stimulation of G-protein linked receptors that increase phospholipase A2 and therefore DAG. A recent study of a transgenic mouse allowing transient cardiac specific overexpression of DAG kinase ζ showed that such overexpression, which lowers DAG levels, prevents ventricular tachyarrhythmia in a HF [198]. This was interpreted by the authors as supporting a role of the TRPC receptors in HF.

The TRPC group of receptors is thought to have other important interactions with other regulatory pathways. Specifically stimulation of these channels and consequent increase in cytosolic Ca^2+^, is thought to lead to increased calcineurin activity and consequent stimulation of NFAT signaling [195–197]. Calcineurin and NFAT, as discussed above, both have important roles in HF. Furthermore, there are DNA sequences in the promoter regions of *TRPC1*, *TRPC3* and *TRPC6* that appear to be NFAT responsive enhancers. While only the TRPC6 sequences have clearly been shown to function as NFAT responsive elements, it is possible that all three of these genes are induced by NFAT signaling. It can be argued, therefore, that TRPC6 and possibly these other two *TRPC* genes are involved in a positive feedback loop with calcineurin and NFAT, with the channels providing cytosolic Ca^2+^ which stimulates calcineurin and NFAT which then leads to the synthesis of more channels [195]. Furthermore, the NO/sGC/cGMP/protein kinase G pathway may down-regulate the TRPC1/TRPC3/TRPC6 channel activities through protein kinase G-dependent phosphorylation of the channel proteins [195], thus providing an additional possible mechanism by which cGMP may act to lower susceptibility to HF.

Transgenic mouse studies demonstrate roles of these channels in HF. Kuwahara *et al*. [199] showed that TRPC6 expression was increased in normal mice by calcineurin/NFAT signaling. Cardiac-specific overexpression of the TRPC6 gene leads to “heightened sensitivity for lethal cardiac growth, and heart failure, an increase in NFAT-dependent expression of beta-myosin heavy chain” and pathologic hypertrophy [199]. “These findings implicate TRPC6 as a positive regulator of calcineurin-NFAT signaling and a key component in a calcium dependent regulatory loop that drives pathologic cardiac remodeling”.

Much of the signaling interaction discussed above in this section, have been studied in four important studies that are not discussed here [200–203].

Wu *et al*. [204] used cardiac specific expression of dominant negative (dn) mutants of TRPC3, TRPC6 and TRPC4 to determine their effects on the development of HF following pressure overload or neuroendocrine agonist infusion. Each of these dominant negative mutants lowered the cardiac hypertrophic response and partially protected from loss of cardiac function. The dnTRPC4 mutation lowered the expression of the normal *TRPC3/TRPC6/TRPC7* genes, demonstrating an unexpected coordination among these channels [204]. These dominant negative mutants each reduced calcineurin/NFAT signaling. They concluded that “TRPC channels are necessary mediators of pathologic cardiac hypertrophy, (acting) in part through calcineurin-NFAT signaling”.

Seth *et al*. [205] showed that *TRPC1* knockout mice do not develop maladaptive cardiac hypertrophy and preserve cardiac function when subject to pressure overload. They conclude that “TRPC1 channels are critical for adaptation to biomechanical stress and TRPC dysregulation leads to maladaptive cardiac hypertrophy and failure”.

Another member of the TRP receptor family that may have cardiac pathophysiological effects, is TRPM4. It has been shown that a presumably specific inhibitor of TRPM4 can have anti-arrhythmia effects [206]. TRPA1 receptors are also implicated in triggering cardiac arrhymias [207,208]. However, it is unclear whether this involves local action of these receptors in the heart or whether this involves changes in parasympathetic innervation [207,208].

## 6. Initiation: via NO/ONOO-Cycle Elements?

The first principle underlying the NO/ONOO-cycle, discussed above, is that “Stressors that initiate the disease should be able to act by raising cycle elements”. It is important, therefore, to determine whether this principle is followed in various stressors initiating cases of HF. Studies relating to this principle on HF are summarized in Table 2.

It can be seen, from Table 2, that each of 19 diverse stressors that can initiate cases of HF or act as important risk factors in HF, can act, in the context of such initiation or exacerbation, to raise multiple NO/ONOO-cycle elements. In addition, each of the 12 elements of the cycle is implicated in the action of multiple initiators.

## 7. Evidence Relating to Occurrence of the 34 Specific Mechanisms in HF

There are 34 mechanisms that underlie the NO/ONOO-cycle mechanism, listed above. Which of these are documented in HF?

#3 predicts that peroxynitrite and its CO_2_ adduct, break down to produce hydroxyl, carbonate and NO_2_ radicals. This is suggested in HF by the tyrosine nitration found in HF [11,13,14,17,19,212,226] which is thought to be produced not by peroxynitrite itself, but rather by NO_2_ and other radical products of peroxynitrite.
#5 predicts that oxidative stress raises NF-κB activity, as has been shown in studies of HF [241–244].
#7 predicts that NF-κB elevation raises cytokine levels, as shown in studies of HF [67–69].
#8 predicts that some cytokines raise NF-κB activity. The cytokine most extensively shown to do so in a general context is TNF-α and TNF-α stimulation of NF-κB has been shown to occur in HF [244,245], confirming part of this prediction. #8 also predicts that cytokines induce iNOS, shown in HF by [19,72].
#9 predicts that iNOS induction produces large increases in NO levels, has been confirmed in HF [80,81,123].
#10 predicts that the plasma membrane calcium-ATPase which pumps Ca^2+^ ions from the cytosol to the surrounding extracellular fluid is inactivated by tyrosine nitration. This has not been looked at in HF; however, the similar SERCA2A enzyme in the sarcoplasmic reticulum, which is similarly susceptible to inactivation by tyrosine nitration, has been shown to be nitrated and inactivated in HF [13,14]. In the myocardium, the SERCA2a inactivation is thought to be the more important of the two. The action of the NMDA receptors in producing apoptosis in HF [192,193] also strongly suggests a Ca^2+^ apoptotic role.
#13 predicts that elevated Ca^2+^ levels in the mitochondrion can cause apoptosis. This is shown through the role of the calcium-dependent protease, calpain in causing apoptosis in HF [173].
#16 predicts BH4 oxidation by peroxynitrite and #17 predicts NOS uncoupling, as a consequence of BH4 oxidation and depletion. Citations [142–149] have each shown BH4 depletion and consequent NOS uncoupling in HF, and a large number of other studies have also shown NOS uncoupling in HF. Studies [142,145,149] have each documented BH4 oxidation, with [149] showing that the oxidation is produced by peroxynitrite. It follows that both mechanisms #16 and 17 are well-documented in HF.
#18, the activation of poly(ADP-ribose) polymerase (PARP) by peroxynitrite leading to extensive poly ADP-ribosylation of chromosomal proteins and NAD/NADH depletion has been shown on several studies of HF [246–249]. These studies also show a causal role of PARP in HF, based on studies of specific PARP inhibitors and also of a *PARP* gene knockout in the mouse [246–249].
#19 predicts that NO binding to cytochrome oxidase in the mitochondrion will produce increased reduction of electron transport intermediates. Such increased reduction has been reported in [37] for coenzyme Q in HF.
#13, 20–22 and 24 all predict elevated superoxide production from the mitochondrial electron transport chain and elevated mitochondrial superoxide levels. Evidence for such elevated superoxide generation from the mitochondrial electron transport chain in HF has been reviewed by Tsutsui *et al*. [54] and reported elsewhere [37].
#22 predicts cardiolipin oxidation and consequent depletion by peroxynitrite. Such cardiolipin oxidation and depletion has been shown to occur in HF [51–53], although this has not been shown to be caused by peroxynitrite.
#26 predicts lowered aconitase via several mechanisms. Such lowered aconitase has been reported to occur in HF [250,251].
#27 predicts xanthine oxidase (oxidoreductase) elevation. This has been reported to be an important source of oxidants in HF [252,253].
#28 predicts that NADPH oxidase activity is elevated in NO/ONOO-cycle diseases. This has been shown in many studies of HF (see, for example [37,42,214–216,219,250]).

It can be seen, from the above, that 17 of predictions on the specific mechanisms proposed for the NO/ONOO-cycle, have substantial supportive evidence in HF. Furthermore there are six other such mechanisms that are direct consequences of the chemistry and biochemistry, notably #s 1, 2, 4, 12, 14 and 15 and therefore do not need to be confirmed because the compounds and/or enzymes involved are known to be elevated in HF. The author is unaware of any counter-examples, where the predominant evidence argues against one of the 34 mechanisms in HF.

## 8. High Level Endothelin-1 and RhoA as Causal Factors of HF: Both Act as NO/ONOO-Cycle Elements

Each of individual studies that provide evidence for a NO/ONOO-cycle etiology, may be interpreted in other ways [1]. These each may confirm one or more predictions of the NO/ONOO-cycle, but the individual confirmations cannot be considered as strong evidence for the cycle as a whole as the etiologic mechanism. Combinations of evidence may or may not be convincing, but single studies or even types of studies are individually not convincing. However, there was one prediction from the hypothesis that pulmonary hypertension is a NO/ONOO-cycle disease that is an exception. If another factor is well documented to be a causal factor of a proposed NO/ONOO-cycle disease, the cycle predicts that the causal factor must be acting as a cycle element. It may be tissue-limited cycle element, but it must be a cycle element in the relevant tissue for the disease, if the disease is a NO/ONOO-cycle disease. In the pulmonary hypertension paper [1], it was shown that two causal factors of that disease, endothelin-1 (ET-1) and RhoA (acting through Rho kinases), both function as NO/ONOO-cycle elements: That is they are each raised in response to NO/ONOO-cycle elements and they each raise NO/ONOO-cycle elements. RhoA activity is increased by NF-κB and also by the inflammatory cytokines TNF-α and IL-13 and also by reactive oxygen species and oxidative stress [1]. RhoA acting through Rho kinases produces increases in peroxynitrite, superoxide and consequent hydrogen peroxide and oxidative stress, raises cytosolic Ca^2+^, lowers BH4 levels producing NO synthase uncoupling, and raises levels of the inflammatory chemokine IL-8 [1].

ET-1 similarly acts as a NO/ONOO-cycle element. ET-1 has been shown to be induced by inflammatory cytokines and by oxidants with such induction also lowered by antioxidants, it is increased by both NF-κB action and also the action of another inflammatory cytokine activated pathway, it is raised by BH4 depletion and also by TRPV1 activity [1]. Thus five NO/ONOO-cycle elements act to raise ET-1 activity. Furthermore, ET-1 action has been shown to elevate, in turn, essentially the entire NO/ONOO-cycle: oxidative stress, superoxide, the peroxynitrite marker 3-NT, NF-κB activity, NO, lowers levels of BH4, stimulates iNOS induction, increases TRP receptor function, increases levels in cytosolic Ca^2+^, increases NMDA activity, produces mitochondrial dysfunction, and increases inflammatory cytokine levels [1].

Both of these, high level ET-1 [42,210,254–259] and RhoA/Rho kinase [259–266] also appear to be causal factors of HF. Consequently, the same logic holds. The fact that each of these is elevated in response to cycle elements and that each raises cycle elements (in the case of ET-1, essentially the entire cycle is elevated [1]), each provides the most convincing compact set of evidence that the NO/ONOO-cycle is the central cause of HF. It should be noted, however, that although high level ET-1 is a probable causal factor in HF, low levels are required for normal myocyte function and even survival [267,268].

## 9. Discussion and Conclusions

The NO/ONOO-cycle is based on 34 well-documented mechanisms, none of which are original with the author. These link the 12 elements of the cycle together into a series of five underlying cycles (Figure 1A–E), each of which amplifies the others through their common elements. These predict an overall cycle in which the 12 elements are each elevated (with the possible exception of NO [9]) and which is robust and difficult to down-regulate because of this amplification. What is original, here, is that by linking all these together, one produces a proposed overall central etiologic mechanism, and the two questions that each of us must wrestle with in regard to HF, is whether that mechanism is viable for HF and, if viable, is it compelling?

Before leaving this issue of originality, Brown, G. C. and colleagues [269,270] proposed a similar vicious cycle mechanism, although their model was limited to brain diseases.

In attempting to analyze the available literature on HF to determine whether it is likely to be a NO/ONOO-cycle disease, it was necessary to grapple with one inescapable fact. The numbers of studies on HF are huge, such that both time and space has limited the author’s ability to review all the relevant parts of this literature. No doubt that there are important and relevant studies in the literature that are not reviewed here. It has been a goal here, not to cherry pick the data, not to select studies that support the point of view being examined here, but rather to review a sufficient diversity of studies such that one could bring a wide range of information to bear on the central question: Is HF a probable NO/ONOO-cycle disease?

A disease that may be proposed to be a NO/ONOO-cycle disease, can be tested by its fit to five principles that are predicted by the cycle mechanism.

The first principle predicts that stressors initiating cases of a NO/ONOO-cycle disease, must be able to raise cycle elements. The data on this are summarized in Table 2, where the literature on 19 initiating stressors of HF is examined. Table 2 shows that each of the 19, *in the context of HF causation*, raises multiple cycle elements. Furthermore, each of the 12 of the cycle elements is raised by some initiating stressors. So the data on initiation of HF go far beyond the prediction, that each initiating stressor should raise at least one cycle element. It can be seen from this, that the HF has an excellent fit to the first principle. However, it must be pointed out that each of the types of evidence reviewed in Table 2 can undoubtedly be explained in other ways. It is only the pattern of evidence, that the reader may or may not find convincing.

Three of the other principles are examined above together, over much of this paper. The second principle predicts that the elements of the cycle must be raised in the chronic disease phase of illness. The third principle predicts that the symptoms and signs of a NO/ONOO-cycle disease must be produced by one or more elements of the cycle. In other words, if the cycle is causal, elements of the cycle must produce whatever correlates, that is, symptoms and signs, are characteristic of the disease. The fifth principle is that for the cycle to be the central cause of a disease, lowering elements of the cycle should be useful in therapy. Another way of looking at such central causality of the cycle is to perform genetic studies (either conventional or transgenic), demonstrating causality of elements of the cycle, not for specific correlates but for the disease as a whole. These three principles have been examined together because many of the relevant studies provide evidence on two or three of the principles.

There are two caveats that should be examined, before summarizing the information on the second principle:

The issue of NO has been very controversial in HF. The NO/ONOO-cycle predicts that there must be sufficient NO to react with superoxide to produce elevated levels of peroxynitrite. However, NO level is not necessarily elevated because one part of the cycle produces lowered BH4 level which produce lower NO synthesis while raising superoxide synthesis [9]. The data on other proposed NO/ONOO-cycle diseases suggest that NO is elevated, so that is probably the expectation here if HF is a NO/ONOO-cycle disease, but that expectation is not a strong prediction of the NO/ONOO-cycle mechanism. What is the situation for NO in HF?

It has often been stated that there is lowered NO bioavailability in HF, but it is not clear that this is correct. Most of the inference that there is lowered bioavailability is based on lower activity of the NO/sGC/cGMP/protein kinase G pathway, a pathway of action that clearly acts to protect from HF, as discussed above. But four mechanisms produced by oxidative stress and peroxynitrite elevation lower the activity of that pathway. While only two of these, heme Fe II iron oxidation of sGC and oxidative stress induction of PDE5 have been shown to occur in HF, the other two may be predicted to occur there based on their demonstrated role in other types of cardiovascular disease and based on the demonstrated role of both oxidative stress and peroxynitrite elevation in HF. Consequently, lowered activity of this pathway does not allow one to infer lowered NO bioavailability. It is also true that BH4 oxidation and consequent lowered levels, lowered eNOS activity and raised ADMA levels may lower NO availability. However, the substantial iNOS induction, possibly raised nNOS, and Ca^2+^ activation of nNOS and eNOS appear to more than counterbalance the changes that would otherwise tend to lower NO bioavailability. Thus the bulk of the evidence is that NO levels are high rather than low and that lowering iNOS or inhibiting NO synthase activity produces improvements in HF, rather than exacerbating the disease. Finally the important causal role of peroxynitrite and consequent oxidative stress, suggests that again, NO is probably high and that lowering it is helpful because such NO lowering also lowers peroxynitrite. The one context where raising NO levels may be helpful in HF, is when this is done by raising BH4 levels, and some evidence is presented in the BH4 section of the paper, suggesting that this is true.

The other caveat concerns measurements of Ca^2+^ levels in failing hearts *vs*. normal hearts through the heart beat cycle. The lowest levels are substantially elevated in HF, but the highest levels are depressed, as one measures Ca^2+^ levels at different times during the heart beat [156,157]. So, does elevated Ca^2+^ level have an important role in HF? It does, based on the roles of three Ca^2+^ cytosolic receptors in HF (Table 1). It also does, based on the fact that elevated NMDA activity and also elevated TRP receptor activity act mainly by elevating cytosolic Ca^2+^ levels, and both of these receptor types have roles in HF.

With those caveats in mind, all 12 of the NO/ONOO-cycle elements are raised in HF, in agreement with the prediction of the second principle of the cycle. While this is confirmation of an important prediction, it is possible that these raised NO/ONOO-cycle elements may have not causal role in HF, that is they may be an epiphenomenon not the central cause of the disease. It is in this context that the third and fifth principles can each be seen to have great importance.

The third principle states that elements of the cycle must produce the correlates (that is symptoms and signs) of the disease, in a NO/ONOO-cycle disease. Table 3 summarizes a series of 87 studies, discussed above, that each provides confirmation of this prediction for HF. In each of these 87 studies, a NO/ONOO-cycle element is thought to be causal, based on studies of specific biochemical/pharmacological agents and/or specific genetic studies (either conventional genetics or, more often, transgenic mouse studies). Each of these studies shows that one or more of the cycle elements are causally linked to one or more correlates of HF. It can be seen from Table 3 that each of the 12 elements of the cycle has roles in producing HF correlates. This argues that the entire cycle is involved in causation of HF, not just some elements of the cycle. Furthermore, a vast array of correlates of HF is linked to specific NO/ONOO-cycle elements. It is difficult to think of any well-established HF correlates that are *not* so linked. While there can be no doubt that each of these individual studies can be interpreted in other ways, it is difficult to avoid the inference, from the pattern of evidence seen in Table 3, that the NO/ONOO-cycle is the central cause of HF. How else can each cycle element be involved in generating so many correlates (symptoms and signs) of HF? How else can so many HF correlates be caused in part by specific NO/ONOO-cycle elements? How else can all of the cycle elements have roles in generating HF correlates?

The NO/ONOO-cycle, as it appears to apply to HF, is considerably more complex than is envisioned in the 34 mechanisms of the cycle. There are many mechanisms that are clearly relevant to HF, many that can be seen from the discussions on NO, Ca^2+^ and TRP receptors, that are not envisioned in the NO/ONOO-cycle. That should not be surprising. The complexities of the biology are almost always greater than the complexity of our theoretical models of it. An additional source of complexities is likely to come from the specialized nature of the myocardium and the complexity of the regulation that is needed for optimal myocardial function. In biology and medicine, the test of a good theory is not whether it is complete or not. The test is: does it make good predictions? This is where our discussion of the five principles and other predictions come into play.

In the discussion of each of the 12 NO/ONOO-cycle elements, earlier in this paper, evidence was presented that each of these 12 has a causal role in terms of generating HF. Raising cycle elements acts to either initiate cases of HF on its own, or at least increase susceptibility to stressors that can initiate cases of HF; lowering cycle elements protect from the development of HF. Each of the 12 elements act in this way and even certain parts of elements, such as specific TRPC channels or specific Ca^2+^ receptors have causal roles in the disease as a whole. There is then, strong support in studies of HF for the fifth principle of the NO/ONOO-cycle. How else can this be explained, unless the NO/ONOO-cycle or something very similar to it is the central cause of HF?

The fourth principle, the primarily local nature of NO/ONOO-cycle diseases, has been largely ignored in this paper. In a sense this is almost trivially obvious in HF. The cardiac remodeling is clearly local. The many histological changes in the failing heart, some related to remodeling and some not, are clearly local. Clearly, the studies of cardiac specific transgenic mice and HF are based on the assumption, that HF is primarily a local disease. This fourth principle also states that some things are not completely local in NO/ONOO-cycle diseases, including elevated cytokines and other inflammatory markers (*i.e.*, C-reactive protein), BH4 depletion and antioxidant depletion. Each of these three not completely local changes has been shown to occur in blood marker studies in HF. The primary local nature of of HF suggests an explanation for the different types of HF. They may differ from one another in the tissue distribution and severity of the NO/ONOO-cycle, in NO/ONOO-cycle independent effects of initiating stressors on cardiac tissues and on progression of the disease over time. This view suggests that the distribution of iNOS or protein bound 3-NT, such as by using specific antibodies, should be studied in the cardiac tissue of different types of HF.

The excellent fit to each of the five principles in HF is important because each fit provides a very different type of evidence for the causality of the NO/ONOO-cycle. But these are not the only important types of evidence supporting a NO/ONOO-cycle role in HF. Of the 34 specific mechanisms underlying the NO/ONOO-cycle exactly half of them occur in HF and six others may also be inferred to occur as well. However, the most important type of additional evidence comes from the roles of RhoA and endothelin-1 as causal factors in HF.

When there is a causal factor in what is proposed to be a NO/ONOO-cycle disease, the cycle mechanism makes a strong prediction about that factor: It must be acting as a, possibly tissue limited, element of the cycle. This is simple logic based on the cycle being the central cause of the disease. Other causal factors must be raised by cycle elements and must, in turn, raise cycle elements. It follows that substantial evidence that because ET-1 and RhoA are each causal elements for HF, means that if HF is a NO/ONOO-cycle disease, these must both function as cycle elements. These are both, then the types of predictions that Karl Popper, the famous philosopher of science, called a “risky predictions”, predictions that are not likely to be made based on any other, unrelated theory but are nevertheless strong predictions of the NO/ONOO-cycle mechanism. The fact that both ET-1 and RhoA function as cycle elements and are both causal elements in HF, as discussed above, each provides a strong, specific and compact set of evidence that HF is a NO/ONOO-cycle disease.

Clearly, the most important implication of any etiologic mechanism is what it implies for therapy of the disease. If one looks at Figure 1A–E, it can be seen that each of the five cycles that make up the overall NO/ONOO-cycle will be expected to reinforce each other, acting by raising the elements that they have in common with each other. Consequently, the overall NO/ONOO-cycle may be predicted to be robust, such that effective treatment and hopefully a cure, requires down-regulation of all five of the underlying cycles. Treating an established case of HF may well be much more difficutly than preventing the disease from occurring in the first case, because of the robust nature of the cycle. It follows from this that the most effective treatment will be either to down regulate each of the five underlying cycles. Alternatively, it may be possible to simply lower peroxynitrite (ONOO-), the only element that is in common to all five. Is there any evidence that either of these approaches can be effective in the treatment of HF? It turns out that there is some evidence for both.

The drug hydralazine, which has been reported to be a fairly strong peroxynitrite scavenger, has been reported to be a useful treatment for HF [27,271–273]. In addition, HF in animals can be lowered by using other agents that lower peroxynitrite [16,38,45]. Would a focus on peroxynitrite lowering, using still more effective peroxynitrite lowering agents than hydralazine, produce still better human HF clinical responses?

There is also an approach that lowers elements in each of the five underlying cycles. Sirtuin-1 (SIRT1) activity acts to lower eight elements of the NO/ONOO-cycle and is expected therefore to lower all five underlying cycles. Resveratrol, which acts here by raising SIRT1, acts to lower inflammatory cytokines and other inflammatory markers, oxidative stress, iNOS induction, peroxynitrite, superoxide (acting in five different ways), lowers mitochondrial dysfunction, lower NF-κB, lowers excitotoxicity including excessive NMDA activity and raises BH4 levels [274–284]. Resveratrol has been suggested previously as an apparently effective agent for the treatment of HF [278,283,284]. Would optimizing this approach produce still better responses in human HF patients?

Is HF a disease of oxidative stress? That is an important question that must be asked in conjuction with the focus of this special issue. The reaction of NO with superoxide to form peroxynitrite should be viewed as the key mechanism switching on the NO/ONOO-cycle [9] and that BH4 depletion may well have a critical role in that process. The importance of that switch, can be seen in part, because of the action of NO in lowering NF-κB activity and also in lowering NMDA activity, with both of these being activated, mostly indirectly, by peroxynitrite. Thus these two key parts of the NO/ONOO-cycle are predicted to be strongly influenced by the peroxynitrite/NO ratio. This study provides other important mechanisms that supports this view in the context of HF and, perhaps more generally in the context of cardiovascular disease. NO acting by stimulation of the sGC/cGMP/protein kinase G pathway can clearly protect from HF and probably from cardiovascular disease generally. However, NO reacting with superoxide to form peroxynitrite has a key role, possibly *the* key role in turning on the NO/ONOO-cycle and causing HF. *Furthermore each of these two pathways acts to turn off the other pathway.* NO/sGC/cGMP/protein kinase G acts to lower superoxide, the compound that has the crucial role in reacting with NO to form peroxynitrite. Peroxynitrite and consequent oxidative stress act to both oxidize and nitrosylate sGC with both of these blocking NO action in activating the synthesis of cGMP. Furthermore, peroxynitrite mediated nitration of a specific tyrosine residue in protein kinase G inactivates that enzyme. And oxidative stress acts to induce PDE5, leading to lower cGMP levels. While it is clear that the NO/sGC/cGMP/protein kinase G pathway is *not completely* blocked in HF, its lowered activity may well produce substantially increased susceptibility to HF, changes that are independent of the more direct roles of NO/ONOO-cycle elements in producing HF. The actions of each of these two NO pathways in lowering the other, adds to the argument that the peroxynitrite/NO ratio may have a *possibly the key* role in the etiology of HF, adding to the considerations that were discussed at the beginning of this paragraph.

In that sense, these interactions among NO, superoxide and peroxynitrite argue that the answer to the question raised by this special issue is yes. HF is a disease of oxidative stress *because the key factor* that determines whether HF develops or not is the peroxynitrite/NO ratio, with the potent oxidant peroxynitrite inevitably raising oxidative stress and acting in large part in that way. However, in another sense the answer should be at least partly no. It is the whole NO/ONOO-cycle that has a role in causing HF, such that each of the 12 elements of the cycle has important roles, roles that in some cases may be direct. In other cases, elements may simply be acting to raise other cycle elements and thus may act through those other cycle elements. In a vicious cycle mechanism, each element of the cycle is both cause and effect and it is at best arbitrary, therefore, to state that two elements, *i.e.*, peroxynitrite and oxidative stress are the cause of HF.

## Figures and Tables

**Table 1:** Roles of Ca^2+^ receptors in heart failure.

Receptor	Finding	Citation
Calpain	Right ventricular overload in the pig, produces both a lowering and aggregation of talin and right ventricular HF. The calpain inhibitor MDL-28170 normalized each of these and may also normalize aggregation of α-actinin and vinculin.	[167]
Calpain	Review: the role of calpains in myocardial remodeling and HF. Calpains may contribute to myocardial hypertrophy and inflammation, through activation of NF-κB. “They play an important role in the fibrosis process, partly by activating transforming growth factor β. They are also implicated in cell death as they cause the breakdown of sarcolemma and sarcomeres.” In addition, “calpains are indeed actively involved in common causes of HF, including hypertension, diabetes, atherosclerosis, ischemia-reperfusion injury, atrial fibrillation, congestive failure and mechanical unloading.”	[168]
Calpain	Study infers that “calpain mediates dystrophin loss and myofibril degradation in doxorubicin-treated rats.”	[169]
Calpain	Title: Calpain inhibition attenuates right ventricular contractile dysfunction after acute pressure overload.	[170]
Calpain	Calpain inhibitors lower the development of cardiac ventricular hypertrophy, an independent risk factor for HF.	[171]
Calpain	Overexpression of calpastatin, a naturally occurring inhibitor of calpain, attenuates myocardial dysfunction in response to endotoxin exposure.	[172]
Calpain	In cardiomyocytes, calpain 1 activates caspase 3 and poly-(ADP-ribose) polymerase (PARP), as well as apoptosis-inducing factor.	[173]
Calpain	Atrial fibrillation is a specific consequence of calpain activity in cardiac muscle	[174]
Calpain	“These results indicate that biochemical markers of cardiomyocyte cell death, sarcomeric disarray, gelsolin cleavage, and TUNEL-positive nuclei, are mediated, in part by calpain and that calpeptin may serve as a potential therapeutic agent…”.	[175]
Calpain	Calpain I produces Ca^2+^-dependent partial proteolysis of calcineurin, forming Ca^2+^/calmodulin-independent calcineurin.	[176]
CaMKII	One target of action of CaMKII in some types of HF, is phosphorylation and consequent loss of activity of Na(V)1.5 sodium channels in cardiac myocytes.	[177]
CaMKII	CaMKII phosphorylates the titin springs. Such “deranged” CaMKII-dependent phosphorylation occurs in HF and “contributes to altered diastolic stress.”	[178]
CaMKII	A mathematical modeling study suggests that lowering CaMKII phosphorylation along with lowering Ca^2+^ leak may be useful in HF therapy.	[179]
CaMKII	Review: CaMKII seems to be involved in both HF and arrythmias and may, therefore be a promising target for therapy.	[133]
CaMKII	CaMKII-dependent phosphorylation increases inner mitochondrial Ca^2+^ uniporter activity, producing lowered ΔPsi_m_ possibly opening the mitochondrial transition pore. CaMKII action may, therefore, have an important role in HF, including lowering mitochondrial function and increasing apoptotic cell death.	[180]
Calcineurin	A study of cardiac hypertrophy in isolated, adult animal hearts. The authors conclude “Although a direct cause-and-effect relationship between NFAT-luciferase activity and pathological hypertrophy was not proven here, our results support the hypothesis that separable signaling pathways regulate pathological *versus* physiological hypertrophic growth of the myocardium, with calcineurin-NFAT potentially serving a regulatory role that is more specialized for maladaptive hypertrophy and heart failure.”	[181]
Calcineurin	In a study of adaptive response to mouse aortic constriction, “Major calcineurin activation, associated with GSK3b inactivation, appeared to engage maladaptive hypertrophy and progression to HF.”	[182]
Calcineurin	Study of cardiac fibroblast proliferation and fibrosis, in response to electrical field exposure. Showed that field exposure acts to raise cytosolic Ca^2+^ via L-type calcium channel activation, leading to calcineurin and NFAT activation, producing fibroblast proliferation and fibrosis.	[183]
Calcineurin	Transgenic mouse carrying a tetracycline-inducible calcineurin gene; gene activation produced robust cardiac growth resembling pathological hypertrophy, followed by systolic dysfunction, fetal gene activation, fibrosis and HF. Each of these was reversed when the gene was inactivated, except fibrosis, which was partially reversed.	[184]
Calcineurin	Angiotensin II and norepinephrine, both of which can produce HF, were shown to activate the calcineurin, NFAT pathway in cardiomyocytes.	[185]

**Table 2:** Initiating stressors in cases of heart failure and NO/ONOO-cycle elements raised by them.

Initiating stressor	Raised NO/ONOO-cycle elements	Citation
Hypertension/pressure overload	Mitochondrial and general oxidative stress, peroxynitrite, superoxide, NF-κB, BH4 depletion	[44,61,62,142–144]
Mouse mitochondrial superoxide dismutase knockout	Superoxide, oxidative stress	[40,41]
Doxorubicin	Peroxynitrite, superoxide, oxidative stress, Ca^2+^ (particularly in the mitochondrion), NF-κB, iNOS, cytokines TNF-α	[11,38,39,66,209]
Homocysteine elevation	NMDA activity, NO, peroxynitrite, Ca^2+^, probable BH4 depletion	[188–190]
Transplantation—severe ischemia-reperfusion	Superoxide elevation, oxidative stress, mitochondrial dysfunction, peroxynitrite	[16]
Endothelin-1 (ET-1)	Superoxide, iNOS, oxidative stress, Ca^2+^	[42,78,210]
Ovariectomy	BH4 depletion and oxidation; superoxide	[145]
Cardiomyocyte-specific NF-κB elevation (transgenic)	NF-κB, cytokine elevation	[63]
Transgenic calcineurin elevation	Ca^2+^, mitochondrial dysfunction, superoxide	[211]
Post-viral, autoimmune?	iNOS induction, peroxynitrite, inflammatory cytokines, NO	[212,213]
Duchenne muscular dystrophy	Ca^2+^, NO, iNOS induction, mitochondrial dysfunction, oxidative stress and elevated levels of several TRPC channels, superoxide	[214–216]
Endotoxin exposure; sepsis	iNOS, NF-κB, cytokines, superoxide, oxidative stress, Ca^2+^, mitochondrial dysfunction, NO, peroxynitrite	[28,79–82,217–222]
Cardiac-specific transgenic iNOS overexpression	iNOS, NO, oxidative stress, mitochondrial dysfunction	[48,83,84]
Tachypacing	iNOS, BH4 depletion, superoxide, peroxynitrite, Ca^2+^, oxidative stress, mitochondrial dysfunction	[146,223–225]
Myocardial infarction	Ca^2+^, oxidative stress, mitochondrial dysfunction, iNOS, peroxynitrite, NF-κB	[70,209,226]
Hypothyroid [227,228]	Oxidative stress, mitochondrial dysfunction, cytokines	[229,230]
Hyperthyroid [227,228]	Oxidative stress, mitochondrial dysfunction	[43,231–234]
Chagas disease	Ca^2+^, mitochondrial dysfunction, NO, cytokines, iNOS, oxidative stress, superoxide	[235–240]
Cytokines (IL-1β, IFNγ & TNF-α)	Cytokines, iNOS, NO, superoxide, peroxynitrite	[19]

The citations listed in Table 2 are limited to studies on these various stressors in the context of initiation of cases of HF. Some of these stressors have a literature on their raising of still other NO/ONOO-cycle elements, but those have been studied in different contexts and are not being considered here. An example of that is endothelin-1 (ET-1) discussed in the following section. In some of the listings above, superoxide involvement is inferred from either a NADPH oxidase role or an angiotensin II role (which acts to induce NADPH oxidase) or a role of oxidants generated in the mitochondria, because these each act through superoxide generation. Many of the Ca^2+^ indications are from evidence of roles of Ca^2+^ receptors.

**Table 3:** Heart failure correlates produced by NO/ONOO-cycle elements.

Citation	Cycle element(s)	HF correlate changes produced by cycle element
[11]	Peroxynitrite and iNOS (both)	MMP activation, lipid peroxidation
[13,14]	Peroxynitrite, oxidative stress	Tyrosine nitration, oxidation, sulfonylation and consequent inactivation of SERCA2a; lowered rate of relaxation
[15]	peroxynitrite	Creatine kinase tyrosine nitration and inactivation; lowered energy storage and utilization in the myocardium
[18]	peroxynitrite	Cardiomyocyte action potential changes; slowed Ca^2+^ cycling
[20]	peroxynitrite	Decreased response to isoproterenol; lessened ability of isoproterenol to increase Ca^2+^ transients or shortening; increased Tyr284 nitration on protein phosphatase 2a; produces effect by decreasing Ser16 phosphorylation on phospholamban
[28]	peroxynitrite	Produces overall increase in protein-bound 3-NT, oxidative stress, NF-κB elevation, TNF-α elevation
[41]	Mitochondrial superoxide	Mitochondrial energy metabolism dysfunction
[44]	Hydrogen peroxide derived from mitochondrial superoxide	Changes in the mitochondrial proteome associated with HF
[45]	Oxidative stress, probably peroxynitrite	Ventricular remodeling; cavity dilatation and dysfunction
[49]	Mitochondrial oxidative stress	Oxidative changes in enzymes involved in mitochondrial ATP synthesis; energy metabolism dysfunction
[50,54]	Mitochondrial oxidative stress	Myocyte hypertrophy, apoptosis, interstitial fibrosis and MMP activation, producing maladaptive cardiac remodeling and failure; oxidative mtDNA damage and lowered mtDNA copy number
[51–53]	Mitochondrial oxidative stress	Cardiolipin peroxidation
[60]	Oxidative stress	Lowered myocardial Akt signaling, increased connective tissue growth factor
[106,107]	Oxidative stress	Oxidation of heme iron in soluble guanylate cyclase, lowered cGMP synthesis
[161,162]	Oxidative stress	Protein oxidation of RyR2, causes Ca^2+^ leakiness
[61]	NF-κB	Fibrosis, cardiomyocyte hypertrophy; MMP-2 activation; decreased fractional shortening
[62]	NF-κB	Fibrosis and associated increased collagen and fibronectin synthesis; increased connective tissue growth factor
[63]	NF-κB	Myocarditis, inflammatory dilated cardiomyopathy, muscle fiber atrophy; dilated ventricles and atria, strong systolic dysfunction and some diastolic dysfunction
[64,65]	NF-κB	Cardiac hypertrophy
[67–69]	NF-κB	Il-1β, TNF-α, IL-6 elevation
[70]	NF-κB	Systolic dysfunction, lowered chamber remodeling, cytokine expression, fibrosis and apoptosis
[72]	Cytokines, NO	Lowered contractility
[73,74]	Cytokine (TNF-α)	Cardiomyopathy [75] Cytokine signaling Cardiomyocyte mortality, contractile dysfunction, ventricular arrhythmia
[19]	Il-1β, TNF-α, IL-6	iNOS, NO, superoxide, peroxynitrite, lowered cardiac function
[69]	IL-6	Fetal gene expression, cardiomyocyte growth
[80]	iNOS	Cardiac contractile dysfunction
[81]	iNOS, NO	TNF-α elevation, oxidative stress, energy metabolism dysfunction
[48,83]	iNOS, NO	Cardiac hypertrophy, ventricular dilatation, interstitial fibrosis, reactivation of the fetal gene expression; reduced contractility, ejection fraction, and cardiac energetics; up-regulation of peroxiredoxins (a possible protective response)
[84]	iNOS	Mild inflammatory cell infiltrate, cardiac fibrosis, hypertrophy, dilatation; bradyarrhythmia
[85]	iNOS, NO	Lowered isoproterenol responsiveness [87] iNOS Cardiac contractile dysfunction
[126]	Mitochondrial dysfunction (caused by mtDNA mutation)	Dilated cardiomyopathy
[130]	Mitochondrial dysfunction	Cardiac hypertrophy, remodeling
[133]	Mitochondrial Ca^2+^ via CaMKII	Mitochondrial transition pore opening; myocyte apoptosis
[134]	Mitochondrial dysfunction	Lowered cardiomyocyte shortening; aberrant Ca^2+^ cycling
[135]	Mitochondrial dysfunction	Systolic dysfunction; hypertrophy
[140]	Mitochondrial dysfunction	Lowered ejection fraction
[142]	BH4 depletion	NOS partial uncoupling, dephosphorylated phospholamban, diastolic dysfunction, impaired relaxation
[143]	BH4 depletion (acting via eNOS partial uncoupling)	Fibrosis, myocyte hypertrophy, fetal gene expression, oxidative stress, peroxynitrite, MMP-2/9 activation
[144]	BH4 depletion	Hypertrophy, fibrosis, NO synthase uncoupling, oxidative stress
[146,149]	BH4 depletion, iNOS	Both have roles in producing atrial fibrillation and probably cardiomyopathy; NO synthase uncoupling
[167–176] (see Table 1)	Ca^2+^ stimulating calpain(s)	Calpain(s) partial proteolysis is thought to: degrade dystrophin, myofibrils, gelsolin, sarcolemma proteins; activate TGF-β, caspase-3, apoptosis inducing factor, NF-κB; aggregate talin, α-actinin and vinculin. These, in turn are thought to contribute to: fibrosis and remodeling, apoptosis, necrosis, hypertrophy, right ventricular dysfunction, atrial fibrillation
[133, 177–180] (see Table 1)	Ca^2+^ stimulating CaMKII	Phosphorylation of titin springs (contributes to diastolic stress, of Na(V)1.5 (changes action potential, stimulates arrythmia), of mitochondrial Ca^2+^ uniporter (lowers ΔPsi, may stimulate opening of mitochondrial transition pore and apoptosis)
[181–185] (see Table 1)	Ca^2+^ stimulating calcineurin	NFAT pathway, leading to maladaptive hypertrophy; systolic dysfunction, fetal gene expression, fibroblast growth and fibrosis
[186]	NMDA	Negative inotropic effects
[187]	NMDA	Sudden cardiomyopathic death
[188–193]	NMDA	MMP-9 elevation; decreased cell shortening, maximal contraction and relaxation rate, decay of Ca^2+^ transient; raised levels of NO, cytosolic Ca^2+^, calpain activity; cardiac arrhythmia and sudden cardiac death; oxidative stress, mitochondrial dysfunction, NO, cytokines, apoptosis
[198]	TRPC3/TRPC6	Ventricular tachyarrhythmia
[199]	TRPC6	Cardiac hypertrophy, calcineurin/NFAT signaling, beta-myosin overexpression, pathologic remodeling
[204]	TRPC3/TRPC6/TRPC4	Pathologic cardiac hypertrophy, calcineurin/NFAT signaling
[205]	TRPC1	Maladaptive cardiac hypertrophy
[206]	TRPM4	Arrhythmia
